# Biochemical, Metabolomic, and Genetic Analyses of Dephospho Coenzyme A Kinase Involved in Coenzyme A Biosynthesis in the Human Enteric Parasite *Entamoeba histolytica*

**DOI:** 10.3389/fmicb.2018.02902

**Published:** 2018-11-30

**Authors:** Arif Nurkanto, Ghulam Jeelani, Takehiro Yamamoto, Takako Hishiki, Yoshiko Naito, Makoto Suematsu, Tetsuo Hashimoto, Tomoyoshi Nozaki

**Affiliations:** ^1^Graduate School of Medicine, The University of Tokyo, Tokyo, Japan; ^2^Graduate School of Life and Environmental Sciences, University of Tsukuba, Ibaraki, Japan; ^3^Research Center for Biology, Indonesia Institute of Sciences (LIPI), Cibinong, Indonesia; ^4^Department of Biochemistry, School of Medicine, Keio University, Tokyo, Japan; ^5^Clinical and Translational Research Center, School of Medicine, Keio University, Tokyo, Japan

**Keywords:** *Entamoeba histolytica*, coenzyme A, gene silencing, metabolome, drug development

## Abstract

Coenzyme A (CoA) is an essential cofactor for numerous cellular reactions in all living organisms. In the protozoan parasite *Entamoeba histolytica*, CoA is synthesized in a pathway consisting of four enzymes with dephospho-CoA kinase (DPCK) catalyzing the last step. However, the metabolic and physiological roles of *E. histolytica* DPCK remain elusive. In this study, we took biochemical, reverse genetic, and metabolomic approaches to elucidate role of DPCK in *E. histolytica*. The *E. histolytica* genome encodes two DPCK isotypes (*Eh*DPCK1 and *Eh*DPCK2). Epigenetic gene silencing of *Ehdpck1* and *Ehdpck2* caused significant reduction of DPCK activity, intracellular CoA concentrations, and also led to growth retardation *in vitro*, suggesting importance of DPCK for CoA synthesis and proliferation. Furthermore, metabolomic analysis showed that suppression of *Ehdpck* gene expression also caused decrease in the level of acetyl-CoA, and metabolites involved in amino acid, glycogen, hexosamine, nucleic acid metabolisms, chitin, and polyamine biosynthesis. The kinetic properties of *E. histolytica* and human DPCK showed remarkable differences, e.g., the Km values of *E. histolytica* and human DPCK were 58–114 and 5.2 μM toward dephospho-CoA and 15–20 and 192 μM for ATP, respectively. Phylogenetic analysis also supported the uniqueness of the amebic enzyme compared to the human counterpart. These biochemical, evolutionary features, and physiological importance of *Eh*DPCKs indicate that *Eh*DPCK represents the rational target for the development of anti-amebic agents.

## Introduction

Coenzyme A (CoA) is an essential cofactor as an acyl group carrier and carbonyl activating group in metabolism that contributes to 9% out of 3,500 cellular activities.^1^ CoA is synthesized by five to six enzymatic reactions with pantothenic acid (vitamin B_5_), *L*-cysteine, and purine/pyrimidine nucleotides as substrates (Genschel et al., 1999). Pantothenate is either produced *de novo*, as in bacteria, archaea, mammals, and plants (Begley et al., 2001; Chakauya et al., 2008; Spry et al., 2008), or alternatively, scavenged by uptake (Saliba et al., 1998; Spry et al., 2008). It was shown that the first and last steps, catalyzed by pantothenate kinase (PanK) and dephospho-CoA kinase (DPCK, EC 2.7.1.24) are rate limiting and allosterically regulated (Leonardi et al., 2005; Hart et al., 2017). Both enzymes utilize ATP as a phosphate donor and are essential in many organisms (Hart et al., 2017).

*Entamoeba histolytica* is a parasitic protozoan, which causes amebiasis in humans. According to the World Health Organization, 50 million people, especially in the tropical countries, suffer from this infection, resulting in estimated 100,000 deaths annually (World Health Organization [WHO], 1997; Ximénez et al., 2009). No effective vaccine has yet been developed, and only metronidazole and its derivatives are the drugs of choice for treatment. It has been demonstrated that metronidazole targets pyruvate:ferredoxin oxidoreductase, which is involved in acetyl CoA production in central energy metabolism. However, their low efficacy against asymptomatic cyst carriers, cases of treatment failure (Hanna et al., 2000; Ali and Nozaki, 2007), and emergence of resistance (Orozco et al., 1985; Johnson, 1993; Samarawickrema et al., 1997) have been reported. Therefore, other rational targets need to be explored to develop new chemotherapeutics against amebiasis.

In *E. histolytica*, it has been previously shown that repression of *panK* gene causes defect in proliferation, and expression of *dpck* genes was transcriptionally upregulated by repression of *panK* gene expression (Nurkanto et al., 2018). This previous study reinforces the premise that the CoA biosynthetic pathway plays a pivotal role in trophozoite proliferation. While the first rate limiting enzyme was well characterized, the metabolic and physiological roles of the enzyme that catalyzes the last step of the pathway remain elusive. DPCK utilizes ATP to phosphorylate dephospho-CoA at the 3′-hydroxyl group of the ribose moiety to generate CoA. The *E. histolytica* genome apparently encodes two isotypes of DPCK (Nurkanto et al., 2018). In the present study, we conducted biochemical characterization of recombinant enzymes. We also undertook reverse genetic and metabolomic approaches to elucidate the physiological importance of *Eh*DPCK in this parasitic protist.

## Materials and Methods

### Organisms, Cultivation, and Chemicals

Trophozoites of the *E. histolytica* clonal strain HM-1:IMSS cl 6 and G3 strains (Bracha et al., 2006) were maintained axenically in Diamond’s BI-S-33 medium at 35.5°C as described previously (Diamond et al., 1978). Trophozoites were continuously maintained in mid-log phase after inoculation of 1–30th to 1–12th of the total culture volume. *Escherichia coli* BL21 (DE3) strain was purchased from Invitrogen (Carlsbad, CA, United States). Lipofectamine and geneticin (G418) were also purchased from Invitrogen. Ni^2+^-NTA agarose was purchased from Novagen (Darmstadt, Germany). All other chemicals of analytical grade were purchased from Sigma-Aldrich (Tokyo, Japan) unless otherwise stated.

### Phylogenetic Analyses of *E. histolytica* DPCK1 and DPCK2

By Basic Local Alignment Search Tool Protein (blastp) searches in public sequence databases, we collected 88 DPCK sequences including two *E.* *histolytica* sequences from non-redundant protein sequences (nr) database of National Center for Biotechnology Information (NCBI^2^) by using *E. histolytica* *Eh*DPCK1 sequence (EHI_040840/XP_648971) as a query to obtain data from representative taxa (Supplementary Table S1). Hit sequences with *E*-value less than 1 × 10^-10^ were selected. The number of selected sequences in each blastp search for a representative taxon was determined based on the number of listed alignments. Sequences were aligned using Muscle program (Edgar, 2004) in SeaView package version 4.6.1 (Gouy et al., 2010). We selected 112 positions of unambiguously aligned by manual and used for phylogenetic analyses. The data matrices for phylogeny were subjected to IQTREE program (Nguyen et al., 2015) to select appropriate models for amino acid sequence evolution. LG + I + Ã4 and LG + Ã4 models were shown to be the best and the second-best models by using the information criterion. Maximum likelihood (ML) analysis implemented in the RAxML program version 7.2.6 (Stamatakis, 2006) was used to infer ML tree based on the LG + Ã4 model. In the bootstrap analysis, heuristic tree search was performed with a rapid bootstrap algorithm option (-*f*) for 100 bootstrap replicates. Bootstrap proportion (BP) values greater than 50 were indicated on the corresponding internal branches of the ML tree drawn by the use of FigTree program Version 1.4.2.^3^

### Prediction of the Tertiary Structure of *Eh*DPCK1 and *Eh*DPCK2

The structure of *Eh*DPCK1 and *Eh*DPCK2 were predicted by homology modeling using Phyre 2^4^ and *Mus musculus* COASY (PDB ID: 2F6R) as a template. Models were generated using one-to-one threading module of Phyre 2. All illustrations were prepared with EzMol version 1.21 (Reynolds et al., 2018).

### Plasmid Construction for Recombinant *Eh*DPCK1 and *Eh*DPCK2

Plasmids to express recombinant *Eh*DPCK1 and 2 proteins were constructed according to the procedures previously described (Sambrook and Russell, 2001). For PCR amplification of *Ehdpck1* and *2* genes, total RNA was extracted from ∼10^6^ trophozoites of *Ehdpck1* and *Ehdpck2* gene-silenced and control transformant strains using TRIzol (Ambion, Life Technologies) reagent as described in the previous study (Chomczynski and Mackey, 1995). DNase treatment was performed using DNase I (Invitrogen) to exclude genomic DNA. RNA quantity was determined by measuring the absorbance at 260 nm with NanoDrop ND-1000 UV-Vis spectrophotometer (NanoDrop Technologies, Wilmington, DE, United States). DNA fragments corresponding to the protein coding region of *Ehdpck1* and *Ehdpck2* gene were amplified from *E. histolytica* cDNA, using oligonucleotide primers: *Ehdpck1* sense (5′-GCCGGGATCCATGAAAAAGATATTTGTTATTGGT-3′), antisense (5′-GCCGGTCGACTTAAAATTTATTTTCATTGAAGTCAA-3′) and *Ehdpck2* sense (5′-GCCGGGATCCATGGTATTTGTTATTGGTATC-3′), antisense (5′-GCCGGTC GACTTAGTTTAAAGTAATTTTTAATTGTT-3′). Underlined letters indicate BamHI and SalI restriction sites. PCR was performed with primeSTAR HS DNA polymerase (Takara) and the following parameters: an initial incubation at 98°C for 30 s for denaturation; followed by the 30 cycles of denaturation at 98°C for 10 s; annealing at 55°C for 30 s; and elongation at 72°C for 1 min; and a final extension at 72°C for 7 min. The PCR fragments were digested with BamHI and SalI, purified with Wizard^®^ SV gel and PCR clean-up system (Promega). The fragments were cloned into BamHI and SalI double digested *pCold^TM^1* histidine-tag vector (Takara) to finally produce *pCold^TM^1-Ehdpck1* and *pCold^TM^1-Ehdpck2*. The nucleotide sequences of the engineered plasmid were verified by sequencing.

### Production and Purification of Recombinant *Eh*DPCK1 and *Eh*DPCK2

Plasmid *pCold^TM^1-Ehdpck1* and *pCold^TM^1-Ehdpck2* were introduced into *E. coli* BL21(DE3) cells by heat shock at 42°C for 1 min. *E. coli* BL21 (DE3) harboring *pCold^TM^1-Ehdpck1* and *pCold^TM^1-Ehdpck2* were grown at 37°C in 100 mL of Luria Bertani medium (Invitrogen) in the presence of 100 μg/mL ampicillin (Nacalai Tesque). The overnight culture was used to inoculate 500 mL of fresh medium, and the culture was further continued at 37°C with shaking at 180 rpm. When *A*_600_ reached 0.8, 0.5 mM isopropyl β-D-thio galactopyranoside (IPTG) was added, and cultivation was continued for another 24 h at 15°C. *E. coli* cells from the induced culture were harvested by centrifugation at 5,000 ×*g* for 20 min at 4°C. The cell pellet was washed with PBS, pH 7.4, re-suspended in 20 mL of the lysis buffer (50 mM Tris–HCl, pH 8.0, 300 mM NaCl, and 10 mM imidazole) containing 0.1% Triton X-100 (v/v), 100 μg/mL lysozyme, and 1 mM PMSF, and incubated at room temperature for 30 min, sonicated on ice and centrifuged at 25,000 ×*g* for 15 min at 4°C. The supernatant was mixed with 1.2 mL of 50% Ni^2+^-NTA His-bind slurry, incubated for 1 h at 4°C with mild shaking. The recombinant enzyme-bound resin in a column was washed three times with buffer A (50 mM Tris–HCl, pH 8.0, 300 mM NaCl, and 0.1% Triton X-100, v/v) containing 10–50 mM of imidazole. Bound protein was eluted with buffer A containing 100–300 mM imidazole. After the integrity and the purity of recombinant protein were confirmed with 12% SDS-PAGE analysis, followed by Coomassie Brilliant Blue (CBB) staining, the sample was dialyzed against a 300-fold volume of 50 mM Tris–HCl, 150 mM NaCl, pH 8.0 containing 10% glycerol (v/v) and the Complete Mini protease inhibitor cocktail (Roche, Mannheim, Germany) for 18 h at 4°C. The purified enzyme was stored at -80°C with 20% glycerol in small aliquots until use.

### Enzymes Assay

*Eh*DPCK activities of recombinant proteins and those in lysates were measured by a coupling assay using ADP Hunter^TM^ Plus Assay kit (DiscoverX, United States) according to the manufacturer’s instructions. Briefly, enzymatic assays were carried out using 50 ng of recombinant *Eh*DPCK1 or *Eh*DPCK2 and two kind of substrates (4–256 μM for dephospho-CoA, 5–100 μM ATP). All reactions were performed in assay buffer containing 15 mM Hepes, 20 mM NaCl, 1 mM EGTA, 0.02% Tween 20, 10 mM MgCl_2_, and 0.1% bovine gamma globulin in black microplate with 20 μL total volume. Plates were incubated for 60 min, added reagents A and B, and then re-incubated for 60 min in room temperature. After added with stop solution, The fluorescent intensity signal was measured using SpectraMax^®^ Paradigm^®^ (Molecular Devices, CA, United States) at excitation/emission wavelengths of 530/590 nm. The kinetic parameters were calculated using the non-linear regression function using the single saturating concentrations of substrates obtained from the GraphPad Prism software (GraphPad Software Inc., San Diego, CA, United States). The experiments were repeated three times in triplicate using protein preparations from three independent extractions, and kinetic values are presented as the means ± SE.

### Production of *Ehdpck1* and *Ehdpck2* Gene-Silenced Strains

Small antisense RNA-mediated transcriptomic gene silencing (Bracha et al., 2006; Zhang et al., 2011) was used to repress gene expression of *Ehdpck1* and *Ehdpck2* genes with some modifications. Briefly, fragments corresponding to a 400 bp long of the open reading frame were amplified by PCR from cDNA using specific of oligonucleotides as follow: *Ehdpck1* sense (5′-CAG**AGGCCT**ATGAAAAAGATATTTGTTATTGGTAT-3′), antisense (5′-AAT**GAGCTC**CAATGGCAATTTCAGGTGA-3′) and *Ehdpck2* sense (5′-CAG**AGGCCT**ATGGTATTTGTTATTGGTATCAC-3′), antisense (5′-AAT**GAGCTC**TTCTAGTTTCCCAGATAACATTTA-3′). These oligonucleotides contained StuI and SacI restriction sites (shown in bold). PCR products were digested with StuI and SacI (New England BioLabs, MA, United States), and ligated into the StuI and SacI double digested psAP2-Gunma (Husain et al., 2011) to construct gene silencing plasmids. The trophozoites of G3 strain were transformed with the empty vector as a control and silencing plasmids by liposome-mediated transfection as previously described (Nozaki et al., 1999). Transformants were initially selected in the presence of 2 μg/mL geneticin with gradually increased up to 10 μg/mL.

### Reverse Transcription PCR

Approximately 1 μg total RNA was used for cDNA synthesis using First-Stand cDNA Synthesis (Superscript^®^ III, Invitrogen) with reverse transcriptase and oligo (dT) primers according to manufacturer’s instructions. The cDNA product was diluted 10-fold and PCR reactions were carried out in 50 μL, using primer pairs: *Ehdpck1* sense (5′-ATGAAAAAGATATTTGTTATTGGT-3′), antisense (5′-TTAAAATTTATTTTCATTGAAGTCAA-3′) and *Ehdpck2* sense (5′-ATGGTATTTGTTATTGGTATC-3′), antisense (5′-TTAGTTTAAAGTAATTTTTAATTGTT-3′). The PCR conditions were as follows: 98°C for 10 s, followed by 25 cycles of denaturation 98°C for 10 s, annealing at 55°C for 30 s, and extension at 72°C for 1 min. The PCR products obtained were resolved by agarose gel electrophoresis.

### Quantitative Real-Time (qRT) PCR

Relative levels of mRNA of the *Ehpank* (EHI_183060), *Ehdpck1* and *Ehdpck2* (EHI_040840 and EHI_155780, respectively) were measured using qRT-PCR. *RNA polymerase II* gene (EHI_056690) was used as a reference. Each 20 μL reaction mixture contained 10 μL of 2× Fast SYBR Green Master Mix (Applied Biosystems, Foster City, CA, United States), 0.6 μL each of 10 μM sense and antisense primers, 5 μL 10× diluted cDNA, and nuclease-free water. PCR was performed using StepOne Plus Real-Time PCR System (Applied Biosystems, Foster City, CA, United States) with the cycling conditions: enzyme activation at 95°C for 20 s, followed by 40 cycles of denaturation at 95°C for 3 s, and annealing extension at 60°C for 30 s. All reactions were carried out in triplicate, including cDNA-minus controls. The amount of the steady state mRNA of each target gene was determined by the ΔCt method with RNA polymerase II as a reference gene (Livak and Schmittgen, 2001). The mRNA expression level of each gene in the transformants was expressed relative to that in the control transfected with *psAP2*.

### Growth Kinetics

Trophozoite cultures were continuously maintained in mid-log phase as described above. Glass tubes (6-mL, Pyrex) containing approximately 5 × 10^5^ cells were placed on ice for 5 min to detach cells from the glass surface. Centrifugation at 500 × g for 5 min at room temperature was conducted to collect cells. After discarding the spent medium, the pellet was re-suspended in 1 mL of BI-S-33 medium. Cell densities were estimated on a hemocytometer. Approximately 10,000 trophozoites were inoculated in 6 mL fresh BI-S-33 medium. Cell density of the cultures were estimated on a hemocytometer every 24 h for 5 days. Cell viability was also measured using WST-1 (Roche, Mannheim, Germany). Two hundred microliter aliquots of cultures were collected at every time point of incubation and transferred to the 96-well plate. The plate was incubated under anaerobic conditions at 35.5°C for 1 h. After the medium was removed, 100 μL of pre-warmed Opti-MEM I (Life Technologies, Grand Islands, NY, United States) containing 10% volume of WST-1 was added to each well, followed by further incubation at 35.5°C for 30 min. The viability of trophozoites was estimated by measuring absorbance at 450 nm by SpectraMax^®^ Paradigm^®^ (Molecular Devices, CA, United States).

### Cell Fractionation and Immunoblot Analysis to Detect the Localization of *Eh*DPCK

Trophozoites of the amoeba transformant expressing *Eh*DPCK1 or 2 fused to human influenza virus hemagglutinin (HA) and the mock transformant, transfected with *pEhEx*-HA, were washed three times with PBS containing 2% glucose. Cells were homogenized and disrupted in homogenization buffer (50 mM Tris–HCl, pH 7.5, 250 mM sucrose, 50 mM NaCl, and 0.5 mg/mL E-64 protease inhibitor) followed by centrifugation at 500 ×*g* for 5 min, and the supernatant was collected to remove unbroken cells in the pellet. The supernatant fraction was centrifuged at 5,000 ×*g* for 10 min to isolate pellet and supernatant fractions. The 5,000 ×*g* supernatant fraction was further centrifuged at 100,000 ×*g* for 60 min to produce a 100,000 ×*g* supernatant and pellet fractions. The pellets at each step were further washed twice with homogenization buffer and re-centrifuged at 100,000 ×*g* for 10 min. Immunoblot analysis was performed using the fractions and anti-HA mouse monoclonal IgG. Anti-CPBF1 (cysteine protease-binding family protein 1) and anti-CS1 (cysteine synthase 1) rabbit antisera were used as organelle membrane and cytosolic markers, respectively.

### Enzyme Activity and CoA Determination From Cell Lysates

Approximately 10^6^ cells cultivated in 25 cm^2^ flasks (Iwaki, Fukushima, Japan) for 48 h were harvested, and the cell number was estimated as described above. The cell pellet was resuspended and homogenized in homogenization buffer (50 mM Tris–HCl, pH 7.5, 250 mM sucrose, and 50 mM NaCl) supplemented with 1 mM PMSF and 0.5 mg/mL E-64 (Peptide Institute, Osaka, Japan) by mechanical homogenization with a glass homogenizer. After kept on ice for 30 min, the suspension was centrifuged at 500 ×*g* for 30 min at 4°C to remove unbroken cells. Enzyme activity in the supernatant was used for enzymatic assay.

Concentrations of CoA in cell lysates were measured using the CoA assay kit (BioVision, CA, United States) according to the manufacturer’s instructions. CoA at 0.05–1 nmol was used to produce a standard curve to determine the CoA concentrations in lysates. Experiments were conducted in triplicate and repeated three times on three different days.

### Intracellular Metabolite Extraction

Intracellular metabolites from 1 × 10^7^ *Ehdpck1* and *Ehdpck2* gene silencing cells were extracted according to the protocol previously described (Jeelani et al., 2012). Internal standards, 200 μM of 2-(*N*-morpholino)-ethanesulfonic acid and methionine sulfone, were added to every sample to ensure that experimental artifacts such as ion suppression did not lead to misinterpretation of metabolite levels.

### Instrumentation of Capillary Electrophoresis–Mass Spectrometry (CE-MS)

Capillary electrophoresis–mass spectrometry was performed as previously described (Kinoshita et al., 2007; Yamamoto et al., 2014). The instrument used was an Agilent CE Capillary Electrophoresis System equipped with an air pressure pump, an Agilent 1100 series isocratic high-performance liquid chromatography pump and an Agilent 1100 series MSD mass spectrometer. We also used a G1603A Agilent CE-MS adapter kit, and a G1607A Agilent CE-MS sprayer kit (Agilent Technologies). G2201AA Agilent ChemStation software for CE-MSD was used for system control and data acquisition.

### CE-MS Conditions for Cationic, Anionic Compounds, and Nucleotides

Cationic nucleotides compounds were carried out on a gas chromatograph capillary, polydimethylsiloxane (DB-1) (50-μm inner diameter × 100-cm total length) (Agilent Technologies). CE separation was using 50 mM ammonium acetate, pH 7.5 as the electrolyte. Samples solution (∼3 nL) was injected at 50 mbar for 30 s, and a positive voltage of 30 kV was applied. All other conditions were the same as in the anionic metabolite analysis. Anions were coated in SMILE (+) capillary 50-μm inner diameter × 100-cm total length obtained from Nacalai Tesque (Kyoto, Japan). The electrolyte was 50 mM ammonium acetate solution (pH 8.5). Sample solution (∼30 nL) was injected at 50 mbar for 30 s and a negative voltage of 30 kV. The capillary voltage was set at 3.5 kV for ESI-MS in the negative ion mode. Deprotonated [M-H] ions were monitored for anionic metabolites of interest.

### Statistical Analysis of Metabolomic Data and Pathway Analysis

For each experimental condition, three independent biological replicates were made and for each biological replicate, two technical replicates were made. All data are shown as means ± SE or the indicated number of experiments. Statistical comparisons were made by Student’s *t*-test. Pathway analysis was conducted using Kyoto Encyclopedia of Genes and Genomes database (KEGG^5^).

## Results

### Identification and Features of Two *Eh*DPCK Isotypes

Two genes encoding *Eh*DPCK were identified from the genome database of *E. histolytica* HM-1:IMSS (HM-1) reference strain^6^: EHI_040840 and EHI_155780, herein designated as *Ehdpck1* and *Ehdpck2* genes, respectively. The open reading frames of the two genes are 621 and 615 bp in length and encode 206- and 204-amino acid long proteins with the calculated molecular mass of 23.9 and 23.1 kDa, respectively. The two proteins exhibit 38% mutual similarity. *Eh*DPCK1 shows 31, 29, and 26% percentage similarity to orthologs from human, *Arabidopsis thaliana*, and *E. coli*, whereas *Eh*DPCK2 shows 34, 29, and 28% identity to the orthologs from human, *A. thaliana*, and *E. coli*, respectively. Human, *A. thaliana*, and *E. coli* only have one functional copy of DPCK and seem to be slightly closer to *Eh*DPCK2 than to *Eh*DPCK1.

**FIGURE 1:**
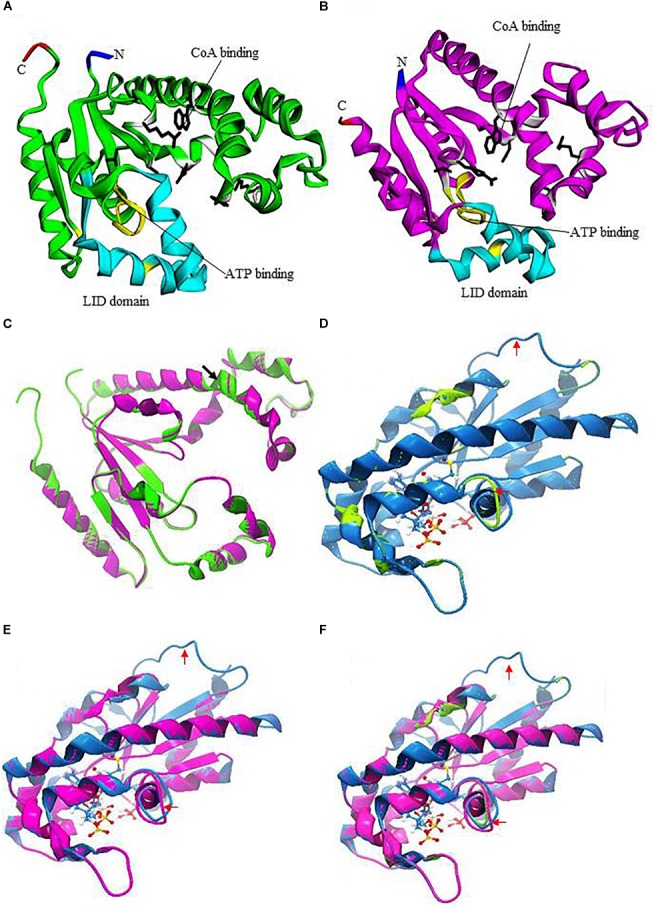
Prediction of three-dimensional structures of *Eh*DPCKs using *Mus musculus* COASY as the Pyre 2 homology model. **(A)** A predicted structure of *Eh*DPCK1 (accession number: XP_648971). The region implicated in the ATP binding is marked in yellow; the side chains of the residues involved in the CoA binding are marked with white ribbons with black sticks; and LID domain is depicted with cyan ribbons. The amino and carboxyl termini are marked in blue and red, respectively. The remaining regions are shown in green. **(B)** A predicted structure of *Eh*DPCK2 (accession number: XP_655761). The region implicated in the ATP binding is marked in yellow; side chains of the residues involved in the CoA binding are marked with white ribbons with black sticks; and LID domain is depicted with cyan ribbons. The amino and carboxyl termini are marked in green and red, respectively. The remaining regions are shown in purple. **(C)** Superimposition of *Eh*DPCK1 (green) and *Eh*DPCK2 (pink). Structures shown in **(C–F)** are rotated 180° around the axis shown in **(A)** of the structure shown in **(A,B)**. Black arrowhead indicated an additional β-sheet in *Eh*DPCK1, but the 3-residue beta sheet is flanked by two alpha helices and thus it is not obvious. **(D)** Superimposition of the *Eh*DPCK1 (green) on *M. musculus* COASY (PDB ID: 2F6R) (blue). ATP is shown with sticks and balls. **(E)** Superimposition of the *Eh*DPCK2 (pink) on *M. musculus* COASY (blue). **(F)** Superimposition of the *Eh*DPCK1 (green) and *Eh*DPCK2 (pink) on *M. musculus* COASY (blue). Red arrows indicate the different area between EhDPCKs and *M. musculus* COASY.

DPCKs are a member of the family of P-loop kinases with an overall topology similar to that of nucleotide monophosphate-binding kinases (Vonrhein et al., 1995; Leipe et al., 2003). DPCKs comprise three domains: the nucleotide-binding domain in parallel β-sheet, the substrate-binding domain in α-helices, and the LID domain. All of these DPCK features are present in *Eh*DPCK (Figures 1A,B). DPCKs generally contain five parallel β-strands flanked by α-helices with ATP-binding site and CoA-binding domain (Obmolova et al., 2001; O’Toole et al., 2003). Based on secondary structure prediction, each monomer of *Eh*DPCKs consist of five to six parallel β-strands flanked by 11 α-helices, which, the domain configuration, differs from 10 α-helices in both *E. coli* and *H. influenza* counterparts. *Eh*DPCK1 and 2 contain the highly conserved P-loop or Walker A sequence motif (GXXXXGKT/S, where X is any residue), which is reported to be involved in the nucleotide binding (Obmolova et al., 2001; Supplementary Figure S1). *Eh*DPCKs P-loop motifs are located in a region between β1 and α1. The amino acid residues involved in the CoA binding (T10, D35, W90, A117, L118, and Q163), and in the ATP binding (G11, I13, G16, K17, R144, and T179) are conserved in *Eh*DPCKs.

*Eh*DPCK1 and 2 show striking similarity based on three-dimensional structure predicted by homology modeling using Phyre 2 (also see below, Figure 1C). *Eh*DPCK1 differs from *Eh*DPCK2 by an additional β-sheet from residue Ile^88^ and Trp^90^, indicated with a black arrowhead (Figure 1C). When compared to *E. coli* DPCK, almost all key residues implicated for CoA binding are conserved in both *Eh*DPCK1 and *Eh*DPCK2 (O’Toole et al., 2003). The only exceptions are the substitution of Val^473^ in human ortholog with Leu^118^ and Leu^117^ in *Eh*DPCK1 and *Eh*DPCK2, respectively. Gly^56^ and Gly^55^ in *Eh*DPCK1 and *Eh*DPCK2, respectively, located between helices α3 and α4 are predicted to be important to maintain structural integrity. Briefly, based on our *in silico* analysis, *E. histolytica* has two genes encoding *Eh*DPCK with relatively low similarity of amino acids identity but share highly conserved amino acids in term of substrates binding.

### Phylogenetic Analysis of *Eh*DPCK

Eighty-eight DPCK sequences from broad taxonomic groups were obtained, aligned, and analyzed (Supplementary Table S1 and Supplementary Figure S1). All major groups of Eukaryota possess putative homologs of DPCK. There are only few other eukaryotes that possess more than one copy of DPCK homologs (e.g., trypanosomes). However, we did not perform further searches for duplicated homologs in other eukaryotic species. While most of the sequences were well aligned throughout the proteins, three eukaryotic homologs (*Homo sapiens, M. musculus*, and *D. melanogaster*) possess an approximately 350 amino acids long N-terminal extension, which corresponds to phosphopantetheine adenylyltransferase domain of the phosphopantetheine adenylyltransferase/DPCK bifunctional enzyme. As shown in an alignment of the selected DPCK sequences from representative taxa (Supplementary Figure S1), the amino acid residues involved in the CoA binding and in the ATP binding are well conserved.

Phylogenetic analyses of 88 sequences on the basis of 112 aligned positions inferred an optimal maximum likelihood (ML) tree shown in Supplementary Figure S2. As a whole, DPCK sequences are highly divergent suggested by long branches. Consequently, no resolution has been inferred for deep branching patterns across all parts of the tree. Neither was inferred the origin of the *Entamoeba* homologs. However, in the genus *Entamoeba*, all DPCK sequences including two isotypes for each species (*E. histolytica, E. nuttalli, E. dispar*, and *Entamoeba invadens*) are monophyletic, although the bootstrap support value was not high (66%). Furthermore, monophyly of copy 1 or copy 2 isotype among the four *Entamoeba* species was supported with 87 and 88% BP values, respectively, suggesting that *DPCK* gene duplication likely occurred in the common ancestor of four *Entamoeba* species, followed by speciation of the four species.

### Structural Comparison of *Eh*DPCK and Its Mammalian Counterpart

We utilized *M. musculus* COASY as a representative mammalian DPCK (the Protein Data Bank ID: 2F6R), as a crystal structure of human DPCK was not available. Human and *M. musculus* COASY are closely related with 87% identity. Molecular modeling using Phyre 2 (see text footnote 4) indicates overall similarities between *Eh*DPCK1 and 2 and also between amebic DPCKs and mammalian counterpart despite low amino acid identity (<24%) (Figures 1D–F). Several areas are remarkably different in structures such as the loop region indicated by red arrows, some residues in CoA binding, and LID domain, suggesting the potential for designing of specific inhibitors against amebic DPCKs.

### Biochemical Characterization of Recombinant *Eh*DPCK1 and *Eh*DPCK2

*Eh*DPCK1 and *Eh*DPCK2 recombinant enzymes were successfully produced using *E. coli* expression system and purified for enzymological characterization. As demonstrated by SDS-PAGE analysis under reducing conditions, followed by CBB staining and immunoblot assay, both enzymes were detected in both the soluble and insoluble fractions. Purified proteins were obtained with a relatively low yield: typically around 0.3 mg of protein, purified from a 500 mL *E. coli* culture (Supplementary Table S2. The purity of recombinant *Eh*DPCK1 and 2 was estimated to be 90∼95% by densitometric scanning of the CBB stained gels after SDS-PAGE (Supplementary Figure S3). Immunoblots also indicate only negligible truncated forms of both *Eh*DPCK1 and 2 were present in the final preparations. The apparent molecular mass of the purified proteins was consistent with the molecular mass of 23.9 and 23.1 kDa for native *Eh*DPCK1 and *Eh*DPCK2, respectively, plus 2.6 kDa corresponding to the histidine tag.

The specific activity of the purified enzymes was estimated as 2.13 ± 0.15 and 2.54 ± 0.26 μmole/min/mg (mean ± SE of triplicates) for *Eh*DPCK1 and *Eh*DPCK2, respectively. *Eh*DPCK1 and *Eh*DPCK2 showed a broad pH optimum between 7 and 9 with the maximum activity at 8 (Supplementary Figure S4). No difference was observed in the pH dependency between *Eh*DPCK1 and *Eh*DPCK2. Kinetic constants for *Eh*DPCK1 and *Eh*DPCK2 were determined by measuring the initial rates obtained with different concentrations of ATP and dephospho-CoA (Table 1). Both ATP and dephospho-CoA exhibited hyperbolic saturation kinetics when assayed over the range of 4–128 μM dephospho-CoA in the presence of 100 μM ATP and in the range of 1–100 μM ATP substrate in the presence of 128 μM dephospho-CoA (data not shown). *Eh*DPCK1 showed the *K*_m_ values of 114 ± 19 and 19.6 ± 1.2 μM for dephospho-CoA and ATP, respectively. *Eh*DPCK2 showed lower *K*_m_ values with 57.9 ± 6.1 and 15.0 ± 2.4 μM for dephospho-CoA and ATP, respectively. Neither *Eh*DCPK1 nor 2 utilized pantothenate as a substrate when the assay was conducted with 100 μM of pantothenate and 80 μM of ATP (<0.02 μmole/min/mg; data not show in tables).

**Table 1:** Kinetic parameters of *E. histolytica* dephospho CoA kinase 1 (*Eh*DPCK1) and dephospho CoA kinase 2 (*Eh*DPCK2).

Enzyme	Substrate	*K*_m_ (μM)	*V*_max_ (μmole/min/mg)	*K*_cat_ (min^-1^)	*K*_cat_/*K*_m_ (min^-1^μM^-1^)
*Eh*DPCK1	Dephospho CoA	114 ± 19	3.71 ± 0.43	88.9 ± 10.3	0.78 ± 0.04
	ATP	19.6 ± 1.2	3.54 ± 0.09	84.8 ± 2.4	4.32 ± 0.23
*Eh*DPCK2	Dephospho CoA	57.9 ± 6.07	2.48 ± 0.15	57.5 ± 3.5	0.99 ± 0.07
	ATP	15.0 ± 2.4	2.71 ± 0.17	62.9 ± 3.7	4.25 ± 0.74

*Assays were performed in the presence 10 mM MgCl2, 15 mM HEPES, 20 mM NaCl, 1 mM EGTA, 0.02% Tween-20, 0.1 mg/mL β-globulins, and 4–256 μM dephospho-CoA to determine kinetic parameters toward ATP or 5–100 μM ATP to determine kinetic parameters toward dephospho-CoA. Reactions were conducted at 37°C at pH 8 for 60 min. Mean ± SE of three replicates are shown*.

Recombinant *Eh*DPCK1 and *Eh*DPCK2 could use various nucleoside triphosphates, such as ATP, CTP, GTP, and UTP, as phosphoryl donors (Table 2). GTP could partially replace ATP for *Eh*DPCK1 and *Eh*DPCK2 (33% of the activity relative to that with ATP). This is a unique feature of *Eh*DPCKs as in general only a few kinases exhibit dual usage of ATP or GTP (Niefind et al., 1999; Becher et al., 2013). *Eh*DPCK1 and *Eh*DPCK2 showed noticeably difference in nucleotide triphosphate preference toward TTP (24.1 and 4.9%, respectively). The effects of metal ions were examined by assaying the activity after the addition of metal salts to the standard reaction mixture (Table 3). The data also suggest that both *Eh*DPCK1 and *Eh*DPCK2 activities are Mg^2+^-dependent. Furthermore, some cations (Zn^2+^ and Cu^2+^) could replace Mg^2+^ with relative activity higher than 50%. No activity was detected when Na^+^ or K^+^ was used instead of Mg^2+^. Both *Eh*DPCK1 and *Eh*DPCK2 showed similar profiles of inhibition by CoA, acetyl-CoA, and malonyl-CoA at relatively high concentrations (Supplementary Figure S5). Taken together from enzymological characterization including kinetic parameters, phosphoryl donor specificities, metal dependence, and inhibitors, *Eh*DPCK1 and 2 showed are both active and show noticeable biochemical differences.

**Table 2:** Phosphoryl donor specificity^a^ of *Eh*DPCK1 and *Eh*DPCK2.

Phosphoryl donor^b^	Relative activity (%)	
	*Eh*DPCK1	*Eh*DPCK2
ATP	100.0 ± 0.0	100.0 ± 0.0
TTP	24.1 ± 2.2	4.9 ± 1.1
GTP	32.6 ± 2.6	33.0 ± 3.8
CTP	15.4 ± 1.8	7.3 ± 1.6
UTP	2.6 ± 0.1	4.5 ± 0.3
None	ND	ND

*^*a*^Assays were performed in the presence 10 mM MgCl_2_, 15 mM HEPES, 20 mM NaCl, 1 mM EGTA, 0.02% Tween-20, 0.1 mg/mL β-globulins and 1 mM dephospho-CoA. Reactions were conducted at 37°C in pH 8 for 60 min. ^*b*^The final concentration used was 0.1 mM. The activity is shown in percentage (%) relative to that toward ATP. ND, not detected. Mean ± SE of three replicates are shown*.

**Table 3:** Effect of metal ions^a^ on the activity of *Eh*DPCK1 and *Eh*DPCK2.

Metal^b^	Relative activity (%)	
	*Eh*DPCK1	*Eh*DPCK2
MgCl_2_	100.0 ± 0.0	100.0 ± 0.0
FeCl_2_	20.9 ± 1.8	12.1 ± 2.7
CaCl_2_	10.5 ± 1.7	1.0 ± 0.5
CoCl_2_	28.3 ± 6.2	40.9 ± 0.2
MnCl_2_	43.8 ± 2.2	53.7 ± 3.1
ZnCl_2_	76.9 ± 0.3	78.3 ± 0.1
NiCl_2_	45.2 ± 0.3	23.0 ± 2.2
CuCl_2_	53.2 ± 5.1	61.5 ± 5.1
LiCl_2_	4.9 ± 0.2	21.7 ± 1.5
NaCl	ND	ND
KCl	ND	ND
None	ND	ND

*^*a*^Assays were performed in the presence 0.1 mM ATP, 15 mM HEPES, 20 mM NaCl, 1 mM EGTA, 0.02% Tween-20, 0.1 mg/mL β-globulins, and 1 mM dephospho-CoA. Reactions were conducted at 37^o^C in pH 8 for 60 min. ^*b*^The cation final concentration used was 5 mM. The activity is shown in percentage (%) relative to that toward MgCl_*2*_. ND, not detected. Mean ± SE of three replicates are shown*.

### Cellular Localization of *Eh*DPCK

Cellular fractionation of the transformant cells expressing HA-tagged *Eh*DPCK2, followed by immunoblot analysis with anti-HA antibody was performed in order to determine localization of *Eh*DPCK2. *Eh*DPCK2 was detected in a wide range of fractions, namely 5,000 ×*g* supernatant and pellet and 100,000 ×*g* supernatant and pellet fractions with the highest amount being detected in the 100,000 ×*g* supernatant fraction, suggesting that *Eh*DPCK2 is located in both cytosol and organelles (or associated with the membrane) (Supplementary Figure S6C). Despite repeated trials, *Eh*DPCK1 with epitope-tags (i.e., HA, Myc, and Flag) were unable to be expressed.

### Phenotypes Caused by Gene Silencing of *Ehdpck1* and *Ehdpck2*

**FIGURE 2:**
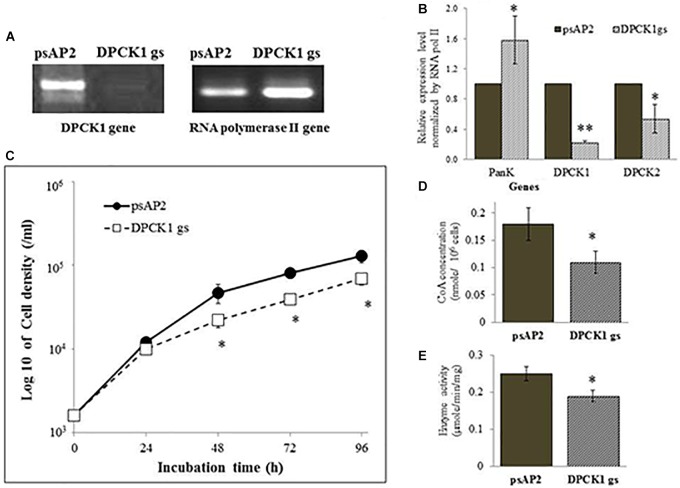
The *dpck* gene expression, cell growth, CoA concentration, and DPCK activity in *Ehdpck1* gene-silenced (gs) strain. **(A)** RT-PCR of *Ehdpck1* and *RNA polymerase II* genes from cDNA of *E. histolytica* transformants. “*psAP2*” indicates G3 strain transfected with mock *psAP-2-Gunma* vector, and “DPCK1 gs” indicates GS strain transfected with *Ehdpck1* gene silencing plasmid. **(B)** Relative levels of gene transcripts encoding *Eh*PanK, *Eh*DPCK1, and *Eh*DPCK2 involved in CoA biosynthesis, estimated by qRT-PCR analysis in *psAP2* and *Ehdpck1* gs transformants. qRT-PCR data were normalized against *RNA polymerase II*, and are shown in percentage relative to the transcript level of each gene in *psAP2* control. **(C)** Growth kinetics of *E. histolytica* transformants in BI-S-33 medium. **(D)** CoA concentrations in cell lysates. **(E)** DPCK activity in cell lysates of *psAP2* and *Ehdpck1* gs transformants. Data shown in mean ± SE. Statistical comparison is made by Student’s *t*-test (^∗^*P* < 0.05, ^∗∗^*P* < 0.01).

**FIGURE 3:**
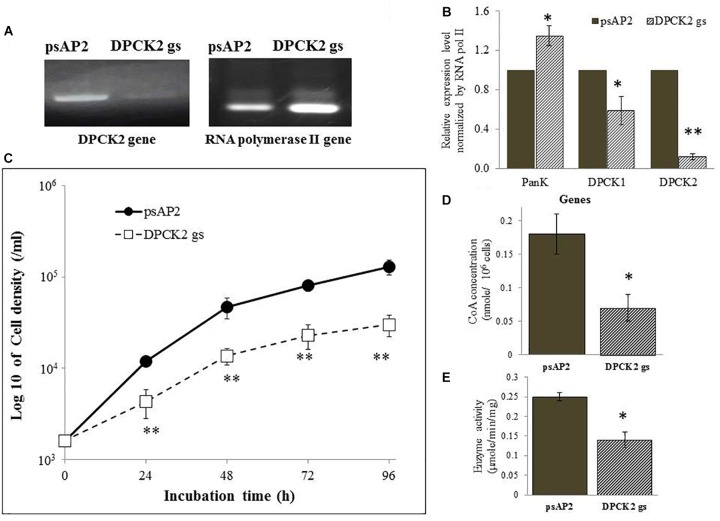
DPCK gene expression, cell growth, CoA concentration, and DPCK activity in *Ehdpck2* gene-silenced strain. **(A)** RT-PCR of *Ehdpck2* and *RNA polymerase II* genes from cDNA of *E. histolytica* transformants. **(B)** Relative levels of gene transcripts encoding *Eh*PanK, *Eh*DPCK1, and *Eh*DPCK2, estimated by qRT-PCR analysis using RNA from *psAP2* and *Ehdpck2* gs transformants. qRT-PCR data were normalized against *RNA polymerase II*, and are shown in percentage relative to the transcript level of each gene in *psAP2* control. **(C)** Growth kinetic of *E. histolytica* transformants in BI-S-33 medium. **(D)** CoA concentrations in cell lysates. **(E)** DPCK activity in cell lysates of *psAP2* and *Ehdpck2* gs transformants. Labels and abbreviations are as in Figure 1.

To better understand the role of *Eh*DPCK in *E. histolytica*, we created a transformant strain where *dpck* gene expression was selectively repressed by small antisense RNA-mediated transcriptional gene silencing. A 400-bp long region of *Ehdpck1* and *Ehdpck2* genes that corresponds to the amino-terminal portion of the protein was used to design gene silencing constructs. Despite low identity at nucleotide and amino acid levels between the two isotypes, gene silencing of *Ehdpck1* gene also affected *Ehdpck2* gene and *vice versa* (Figures 2A,B, 3A,B). Gene silencing of expression *Ehdpck1* gene caused 77 ± 1.2 and 46 ± 18.9% reduction of *Ehdpck1* and *Ehdpck2* transcripts, respectively, while gene silencing of *Ehdpck2* gene caused 41 ± 14.8 and 88 ± 2.9% reduction of *Ehdpck1* and *Ehdpck2* transcripts, respectively. There was no change in the transcript level of irrelevant genes such as NAD(H) kinase (EHI_151920), mitosomal membrane protein-Tom40 (EHI_104420), and cysteine protease binding protein 2 (EHI_087660) (Supplementary Figure S7), suggesting the specificity of *Ehdpck*s silencing. Despite only 45% amino acid identity at the amino-terminal portion of *Eh*DPCK1 and 2, the first 400 bp of *Ehdpck1* and *2* genes show 53% identity at the nucleotide level. Moreover, most of the conserved domains including the ATP-binding domain is located within the first 100 amino acids in both *Eh*DPCK1 and 2. Therefore, this high similarity probably caused cross-repression of gene expression by antisense small RNA-mediated transcriptional silencing in our experiment. Interestingly, gene silencing of either *Ehdpck1* or *Ehdpck2* gene caused upregulation of *EhPanK* transcript (Figures 2B, 3B), which may indicate compensation for *Ehdpck* gene silencing.

*Ehdpck1* and *Ehdpck2* gene-silenced (gs) strains showed significant growth defect in normal growth medium (Figures 2C, 3C and Supplementary Figures S6A,B, by cell count and WST-1 growth assay, respectively). The level of growth inhibition by *Ehdpck2* gene silencing was more severe compared to that by *Ehdpck1* gene silencing; the percentage growth at 96 h of *Ehdpck1* gs and *Ehdpck2* gs trophozoites was 53.8 ± 9.2% and 23.1 ± 6.2% (mean ± SE of triplicates) of the control transformant, respectively. Cells lysates from *Ehdpck1* gs strain contained 38.9 ± 7.1% lower level of CoA (Figure 2D) and approximately 24.0 ± 2.0% less *Eh*DPCK activity (Figure 2E). The effects of *Ehdpck2* gene silencing on the reduction of DPCK activity and CoA concentrations in cell lysates were more severe than those by *Ehdpck1* gene silencing: 61.1 ± 7.1% decrease in the CoA concentration (Figure 3D) and 44.0 ± 6.2% decrease in *Eh*DPCK2 activity (Figure 3E) by *Ehdpck2* gene silencing. Altogether, these results suggest that both *Eh*DPCK isotypes play important roles in cell growth and the role played by *Eh*DPCK2 is more important than that of *Eh*DPCK1. In our study, the repression level of both *Ehdpck1* and *Ehdpck2* gene transcripts by gene silencing was not complete; residual expression was also observed in the silenced strains. This likely contributes the poor but viable cultures of these gene-silenced strains. The fact that we also failed to create *Ehdpck*s gene-silenced lines in three additional attempts is also consistent with the presumed essentiality of these genes.

### Metabolomic Analyses of *Ehdpck1* and *Ehdpck2* Gene-Silenced Strains

We performed metabolomics analyses of the *Ehdpck1* and *Ehdpck2* gs strains. The capillary electrophoresis–mass spectrometry (CE-MS)-based quantitation systems were used both in cation and anion modes to identify >110 metabolites which include precursors, intermediates, and end products of central carbon metabolism, biosynthetic and catabolic intermediates of amino acids, sugars, nucleic acids, and lipids. All data were presented as normalized by cell number (per 1 × 10^6^ cells), as commonly accepted and used in many studies. The levels of some metabolites were changed in both directions (i.e., increase and decrease) in response to *Ehdpck1* and *Ehdpck2* gene silencing (Supplementary Table S3). However, all of the statistically significant changes in the concentrations of metabolites in both *Ehdpck1* and *Ehdpck2* gene silencing were decrease, not increase, relative to the metabolite levels in the control strain.

**FIGURE 4:**
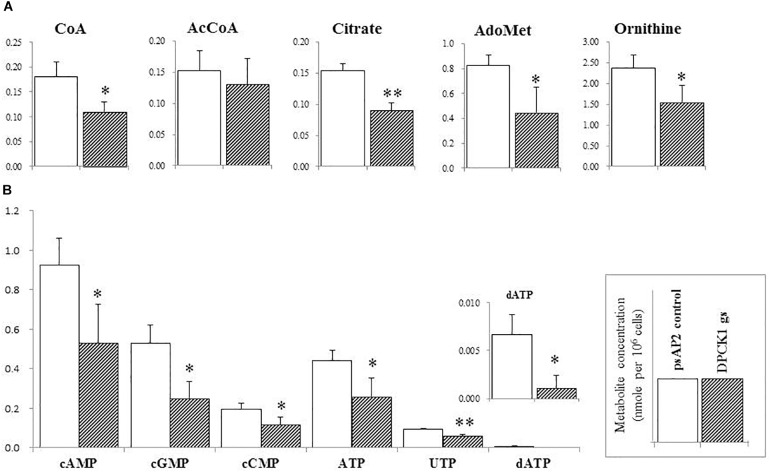
The levels of representative intermediary metabolites affected by *Ehdpck1* gene silencing. **(A)** Note decrease in the concentrations of coenzyme A (CoA), acetyl-CoA (AcCoA), citrate, ornithine, and *S*-adenosyl-L-methionine (AdoMet). **(B)** Depletion of nucleotides. Data are shown in mean ± SE. Statistical comparison is made by Student’s *t*-test (^∗^*P* < 0.05, ^∗∗^*P* < 0.01).

**FIGURE 5:**
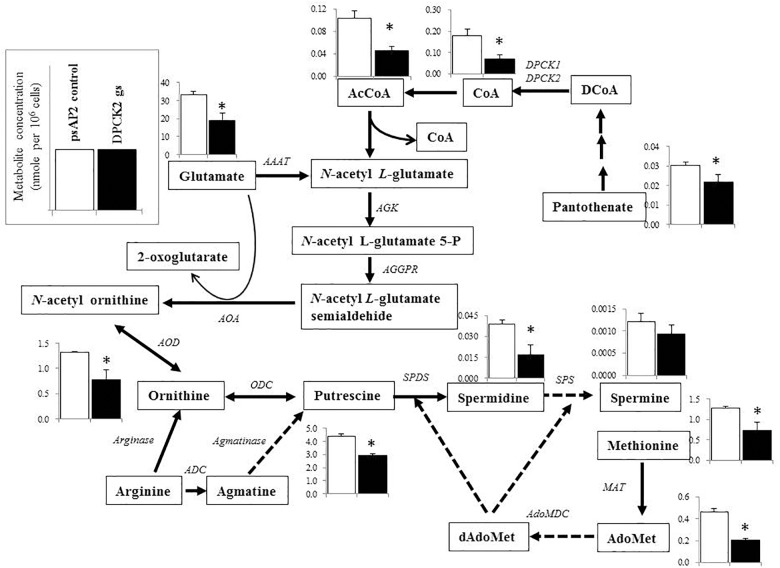
Metabolic profiles of amino acids, polyamines, and associated metabolites affected by *Ehdpck2* gene silencing. Broken lines indicate genes whose encoding enzymes known to catalyze the steps are likely absent in the genome. Abbreviations: ND, not detected; CS, citrate synthase; AAAT, amino-acid *N*-acetyltransferase; AGK, acetylglutamate kinase; AGGPR, *N*-acetyl-L-glutamyl-phosphate reductase; AOA, acetylornithine aminotransferase; AOD, acetylornithine deacetylase; ODC, ornithine decarboxylase; ADC, arginine decarboxylase; MAT, methionine adenosyltransferase, AdoMet, *S*-adenosyl-L-methionine; dAdoMet, *S*-adenosylmethioninamine; MAO, monoamine oxidase; AdoMDC, S-adenosylmethionine decarboxylase; SPDS, spermidine synthase; SPS, spermine synthase. Short and successive arrows indicated multiple steps in the corresponding pathway. Data are shown in mean ± SE. Statistical comparison is made by Student’s *t*-test (^∗^*P* < 0.05).

**FIGURE 6:**
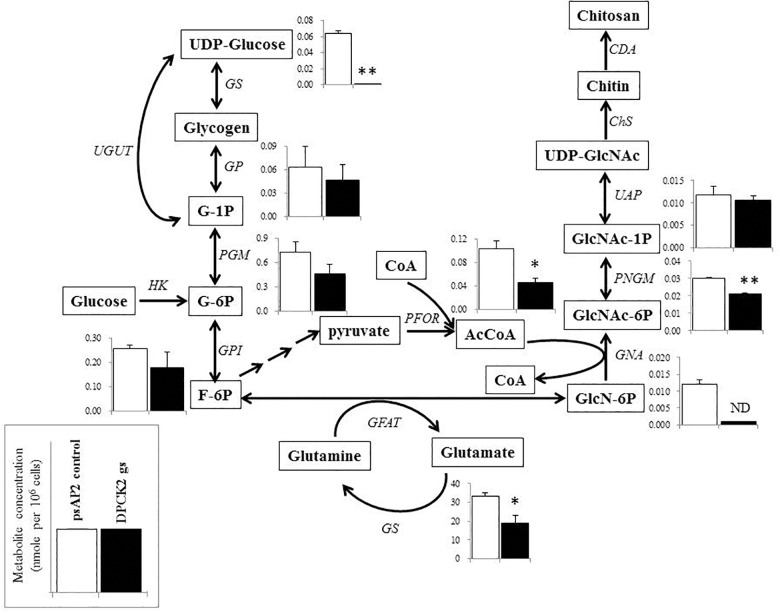
Metabolic profiles of hexose phosphates, glycogen metabolism, chitin biosynthesis, and associated metabolism affected by *Ehdpck2* gene silencing. Abbreviations: G-1P, glucose 1-phosphate; G-6P, glucose 6-phosphate; F-6P, fructose 6-phosphate; GlcN-6P, glucosamine 6-phosphate; GlcNAc-6P, *N*-acetylglucosamine 6-phosphate; GlcNAc-1P, *N*-acetyl glucosamine 1-phosphate; UDP-GlcNAc, UDP-*N*-acetylglucosamine; GS, glycogen synthase; GP, glycogen phosphorylase; UGUT, UTP-glucose-1-phosphate uridyltransferase; PGM, phosphoglucomutase; GPI, glucose-6-phosphate isomerase; HK, hexokinase; GFAT, glutamine:fructose-6-phosphate aminotransferase; GS, glutamine synthetase; PFOR, pyruvate ferredoxin oxidoreductase; GNA, glucosamine-phosphate *N*-acetyltransferase; PNGM, phosphoacetylglucosamine mutase; UAP, UDP-*N*-acetylgalactosamine diphosphorylase; ChS, chitin synthase; CDA, chitin deacetylase; ND, not detected. Short and successive arrows indicated multiple steps in the corresponding pathway. Data are shown in mean ± SE. Statistical comparison is made by Student’s *t*-test (^∗^*P* < 0.05, ^∗∗^*P* < 0.01).

**FIGURE 7:**
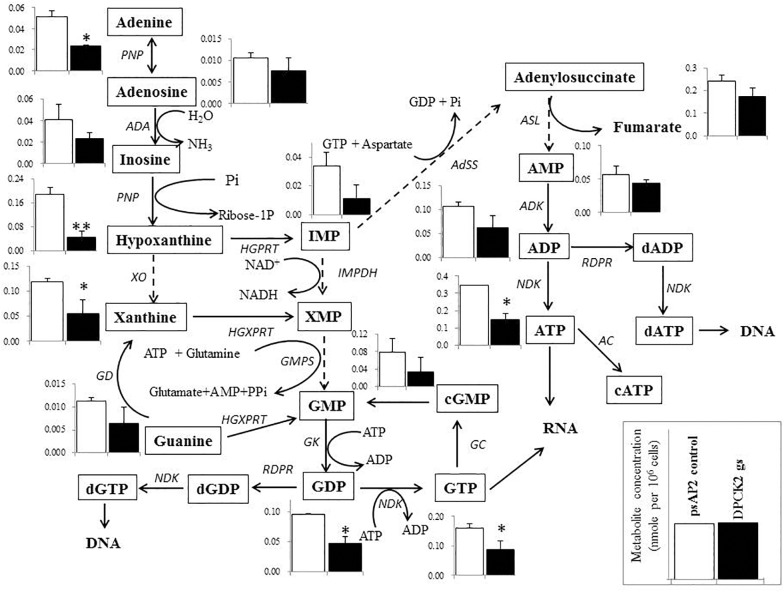
Metabolic profiles of purine metabolism affected by *Ehdpck2* gene silencing. Broken lines indicate genes whose encoding enzymes known to catalyze the steps are likely absent in the genome. Abbreviations: PNP, purine nucleoside phosphorylase; ADA, adenosine deaminase; XO, xanthine oxidase; HGPRT, hypoxanthine-guanine phosphoribosyltransferase; HGXPRT, hypoxanthine-guanine-xanthine phosphoribosyltransferase; IMPDH, inosine 5’-monophosphate d*Eh*ydrogenase; GMPS, guanosine monophosphate synthetase; GK, guanylate kinase; NDK, nucleoside diphosphate kinase; RDPR, ribonucleoside diphosphate reductase; GC, guanylate cyclase; AdSS, adenylosuccinate synthetase; ASL, adenylosuccinate lyase; ADK, adenylate kinase; AC, adenylate cyclase. Data are shown in mean ± SE. Statistical comparison is made by Student’s *t*-test (^∗^*P* < 0.05, ^∗∗^*P* < 0.01).

Besides decrease in the CoA concentration mentioned above, *Ehdpck1* gene silencing caused decrease only in citrate, ornithine, some nucleic acids, and *S*-adenosyl-L-methionine (Figure 4). On the other hand, *Ehdpck2* gene silencing caused decrease in a wider range of metabolites than *Ehdpck1*, such as pantothenate, acetyl-CoA, glutamate, ornithine, putrescine, spermidine, methionine, and *S*-adenosyl-L-methionine, which were mapped to metabolites involved in ornithine and polyamine biosynthesis in KEGG maps (Figure 5). *Ehdpck2* gene silencing also caused decrease in some (five of nine metabolites measured) metabolites involved in glycogen/glucuronate and chitin metabolism, such as UDP-glucose, glutamate, and *N*-acetylglucosamine 6-phosphate (Figure 6). Interestingly, some (six of 14 metabolites measured) of the intermediate metabolites in purine metabolism were also decreased by *Ehdpck2* gene silencing (Figure 7): in particular adenine, hypoxanthine, xanthine, GDP, GTP, and ATP. Metabolite changes caused by *Ehdpck1* gene silencing in ornithine and polyamine biosynthesis, glycogen/glucuronate, chitin, and purine metabolisms were presented in Supplementary Figures S8–S10. Taken together, our metabolomics data showed that *Ehdpck2* gene silencing more severe affected overall metabolisms than that of *Ehdpck1*.

## Discussion

### Suggested Specific Roles of *Eh*DPCK Isotypes

Our previous study on *E. histolytica* PanK, the enzyme that catalyzes the first step of CoA biosynthesis, clearly demonstrated that this enzyme was important for growth of *E. histolytica* trophozoites. In this study, we have shown that both of two *Eh*DPCKs, *Eh*DPCK1 and *Eh*DPCK2, play an indispensable, likely non-overlapping role in CoA biosynthesis. A line of evidence supports that both enzymes are involved in CoA biosynthesis in *E. histolytica*. First, *E. coli-*produced recombinant enzymes show comparable specific activity and kinetic parameters. Second, *Ehdpck1* and *Ehdpck2* gene silencing caused decrease in *Eh*DPCK activity and intracellular CoA concentrations, as well as decrease in cell growth. Most eukaryotic organisms possess a single copy of *dpck* gene with some exceptions including *Trypanosoma* (TriTrypDB^7^) and *Entamoeba* (this study). In a rodent *Plasmodium* species, which has only one copy of DPCK, it has recently been reported that DPCK is essential and indispensable for development of blood stage parasites (Hart et al., 2017). Human infecting *Plasmodium* species (*P. falciparum* and *P. vivax*) also have a single copy of *dpck* gene.

In this study, we have shown that *Entamoeba*, where two copies of *dpck* are present, gene silencing of *Ehdpck2* produced more severe effects on both the metabolite profile and growth, compared to that of *Ehdpck1*. Our previous microarray analysis of *E. histolytica* trophozoites showed that the *Ehdpck2* transcript level is higher than that of *Eh*DPCK1, approximately fivefold in HM-1:IMSS cl6 strain (Penuliar et al., 2012, 2015) and 11-fold in G3 strain (Furukawa et al., 2012, 2013; Nakada-Tsukui et al., 2012). Furthermore, based on our previous transcriptomic analysis of encystation (differentiation of the trophozoite into the cyst) of *E. invadens* (De Cádiz et al., 2013), which is the amebic encystation model (Donaldson et al., 1975; Kojimoto et al., 2001; Stanley, 2003; Chia et al., 2009), we found that both of the *E. invadens* genes encoding DPCK1 and DPCK2 were expressed during encystation (Supplementary Figure S11). *E. invadens dpck2* gene was expressed at comparable levels throughout encystation. In contrast, *E. invadens dpck1* transcript was dramatically increased (∼30-fold) during encystation. These data suggest that these isoenzymes play specific roles during proliferation and stage conversion. *dpck1* is likely involved in encystation in *E. invadens*. However, gene silencing of *Ehdpck1* and *Ehdpck2* caused comparable, though slightly more pronounced in *Ehdpck2* gs strain, growth and metabolic defects. Our results indicate that *Eh*DPCK2 plays a primary role in the proliferative trophozoite stage and *Eh*DPCK1 may have a specific role during developmental stage transition, which needs to be examined in the future.

### Uniqueness of *Eh*DPCK

Two amebic DPCK isotypes, *Eh*DPCK1 and *Eh*DPCK2, showed comparable specific activity and substrate affinity despite only 38% mutual identity. When the *K*_m_ values of amebic DPCKs were compared with the human counterpart, phosphopantetheine adenylyltransferase and DPCK bifunctional enzyme (Aghajanian and Worrall, 2002) from human has an approximately 10-fold higher *K*_m_ value toward dephospho-CoA and a 10-fold lower *K*_m_ value toward ATP (5.2 and 192 μM, respectively). These data indicate significant biochemical and structural differences exist between amebic and human DPCK and also suggest a possibility of discovering or designing parasite-specific DPCK inhibitors. ATP production in *E. histolytica* relies solely on substrate-level phosphorylation during glycolysis because this parasite lacks the enzymes and machineries involved in the Krebs cycle and oxidative phosphorylation and thus is unable to produce ATP as efficiently as aerobic organisms (McLaughlin and Aley, 1985; Reeves, 1985). It is thus plausible that DPCK from *E. histolytica* is able to have higher affinity to ATP than human counterpart. Moreover, our results suggest that both *Eh*DPCK1 and *Eh*DPCK2 activity was regulated biochemically not only by the end products of this pathway, CoA, but also its thioesters, acetyl CoA and malonyl-CoA through feedback inhibition.

### Domain Structures

Dephospho-CoA kinase has three domains: the nucleotide binding, the dephospho-CoA binding, and the LID domains (Obmolova et al., 2001). In *E. coli*, two highly conserved prolines (Pro^90^ and Pro^134^) are predicted to be essential for the lid flexibility. These residues are located between the nucleotide binding and dephospho-CoA binding domains. However, these residues are only partially conserved in *Eh*DPCK1 (Pro^138^) and *Eh*DPCK2 (Pro^90^), indicating that the lid flexibility of DPCK is different between *E. histolytica* and its bacterial ortholog. A lysine residue in the GXXXGKS motif is also one of the conserved motifs of DPCKs among organisms, and known to be essential for the binding of ATP. Thr^10^ for *Eh*DPCK1 and Thr^8^ for *Eh*DPCK2 are probably involved in dephospho-CoA binding, whereas Asp^35^ in *Eh*DPCK1 and Asp^33^ for *Eh*DPCK2 are involved in activation of the 3′-OH group of the ribose for the attack on the β-phosphate of ATP, as previously reported in bacteria (Obmolova et al., 2001; O’Toole et al., 2003). Leu^85^ in *Eh*DPCK1 and Ile^84^ in *Eh*DPCK2, both of which are also predicted to be localized to the CoA-binding site, are also conserved. Asn^91^ in *Eh*DPCK1 is replaced with Pro^90^ in *Eh*DPCK2; the corresponding residue Pro^90^ in *E. coli* is located at a hinge and implicated for the movement of the CoA-binding domain during catalysis, suggesting a potential difference in a catalytic process between *Eh*DPCK1 and 2.

### Metabolic Disturbance Caused by *Ehdpck* Gene Silencing

Metabolomics analysis has been widely used to complement genomics, transcriptomics, and proteomics analyses (Dumas, 2012; Castro-Santos et al., 2015), and to provide a comprehensive view on the responses to the environmental changes (Fuhrer and Zamboni, 2015), a metabolic-responsive profile (Holmes et al., 2008; Husain et al., 2011), and also to validate target for drug development (Krug and Müller, 2014; Mastrangelo et al., 2014). As expected, *dpck* gene silencing (particularly gene silencing targeting *Ehdpck2*) caused decrease in the level of CoA and acetyl-CoA levels. Acetyl-CoA occupies a crucial position in metabolisms for all living organisms, and serves as an essential metabolic intermediate, a precursor of many anabolic reactions, also a donor of protein acetylation and allosteric enzymatic regulator (Choudhary et al., 2014). Our metabolome data have also shown the disturbance, mostly decrease, in the broad range of related metabolites, in particular those involved in ornithine and polyamine biosynthesis. Ornithine is an important precursor of polyamines and essential for proliferation (Bacchi and Yarlett, 1993; Müller et al., 2001; Heby et al., 2003; Wallace et al., 2003). Polyamine metabolism was targeted to develop drugs against *Trypanosoma* (Begolo et al., 2014) and *Leishmania* (Das et al., 2016). In *P. falciparum*, it has been known that many cellular processes, such as DNA replication, transcription and translation are polyamine dependent and the cellular levels of polyamines reach their peak during the intra-erythrocytic development (Assaraf et al., 1987). Our metabolomics analysis has shown that *Ehdpck2* gene silencing significantly reduced the levels of many intermediates in the polyamine pathway, while *Ehdpck1* gene silencing showed decrease to a lesser extent (not statistically significant). Citrate was significantly decreased by *Ehdpck1* gene silencing, which was not found in *Ehdpck2* gs strain. Citrate is an important intermediate metabolite derived from the condensation of acetyl CoA and oxaloacetate in the Krebs cycle. However, since Krebs cycle is absent in *E. histolytica*, the role of citrate remains elusive and needs future investigation.

The levels of metabolites in hexose metabolism were also decreased by *Ehdpck2* gene silencing (Figure 6). In particular, metabolites involved in the hexosamine-amino acid amide transfer and chitin biosynthesis, such as glucosamine 6-phosphate and *N*-acetyl-D-glucosamine 6-phosphate, significantly decreased compared to control (*P* = 0.008). However, it is not well understood how glucosamine 6-phosphate was completely deprived by *Ehdpck2* gene silencing. The decrease in glucosamine 6-phosphates was apparently associated with the decreased level of glutamate in *Ehdpck2* gs strain, via the reaction converting D-fructose-6-phosphate/L-glutamate and D-glucosamine-6-phosphate and L-glutamine. On the other hand, the decrease in *N*-acetyl-D-glucosamine-6-phosphate was likely caused by the decrease in acetyl-CoA as *N*-acetyl-D-glucosamine-6-phosphate is generated from a combination of D-glucosamine-6-phosophate and acetyl-CoA. Glucosamine 6-phosphate and *N*-acetyl-D-glucosamine 6-phosphate are also important intermediates for chitin biosynthesis. Chitin is important and present as the major component of cell wall in cyst and oocyst stages of many protozoan parasites (Chávez-Munguía et al., 2007) including *E. histolytica* (Arroyo-Begovich and Carabez-Trejo, 1982). Chitin biosynthesis was also proposed as a parasite-specific drug target as it is absent in humans (Spindler et al., 1990). Interestingly, reduction in the intermediates in the chitin biosynthetic pathway was observed only in *Ehdpck2* gs strain, but not in *Ehdpck1* gs strain. Although statistically insignificant, several metabolites in the pathway were increased in *Ehdpck1* gs strain: glucose-1-phosphate, glucose-6-phosphate, *N*-acetyl-D-glucosamine-1-phosphate, and *N*-acetyl-D-glucosamine-6-phosphate, suggesting that the roles of *Eh*DPCK1 and *Eh*DPCK2 in chitin metabolism are different.

Many nucleic acids were also decreased in both *Ehdpck1* and *Ehdpck2* gs strains (Figures 4, 7 and Supplementary Table S3). This may be explained at least in part by the decrease in polyamine synthesis (Figure 5). Polyamines can interact with macromolecules such as nucleic acids and are required during nucleic acids packaging (Ruan et al., 1994). Also, the interaction between polyamines and nucleic acids can affect structure and stability of DNA (Gosule and Schellman, 1976; Raspaud et al., 1999; van Dam et al., 2002). Moreover, most of the intermediary metabolites in purine metabolism in *Ehdpck2* gs strain were also reduced (Figure 7). Importantly, these changes were specific to *Ehdpck2* gene silencing. In *Ehdpck1* gs strain, several purines and intermediates including adenine, guanine, IMP, and ADP, were increased compared to control. Purine metabolism is very important for DNA and RNA synthesis, nucleotide-dependent enzyme reactions, as chemical energy sources (e.g., ATP, GTP), intracellular second messengers (e.g., cAMP), and switch signals (e.g., GDP, GTP) for metabolic and gene regulation (Hyde, 2007). However, a majority of parasitic protists including *E. histolytica* (Lo and Wang, 1985) are incapable of *de novo* synthesis of purines and rely on salvage from the host. Hypoxanthine, guanine, xanthine, adenine, and adenosine must be imported from the hosts or media via nucleoside transporters described in *P. falciparum* (Carter et al., 2000), *Toxoplasma gondii* (De Koning et al., 2003), and *Trypanosoma brucei* (Sanchez et al., 2002). Interestingly, while most of the above-mentioned purines were decreased in *Ehdpck2* gs strain, hypoxanthine was most dramatically decreased (Figure 7). Hypothanthine is regarded as the key precursor of the other purines. For instance, degradation of hypoxanthine by added xanthine oxidase in the culture medium strongly inhibits *in vitro* growth of *P. falciparum* (Berman et al., 1991). Purine metabolic pathways were proposed as promising targets for novel drug development against infections caused by *P. falciparum* (Cassera et al., 2011), *T. gondii* (De Koning et al., 2003), and *Mycobacterium tuberculosis* (Parker and Long, 2007).

### Regulation of CoA Biosynthesis

The *Ehdpck2* gene silencing caused reduction of CoA and also led to decrease in pantothenate, which can be explained by either high consumption of pantothenate or decrease in pantothenate uptake. Most eukaryotes including *E. histolytica* are unable to synthesize pantothenate and rely on uptake of exogenous pantothenate. Our qRT-PCR data indicate that the decreased level of CoA by *Ehdpck1* or *Ehdpck2* gene silencing led to the increase in steady-state mRNA level of *Ehpank* gene encoding the protein that catalyzes conversion of pantothenate to 4′-phosphopantothenate. This was also confirmed by the observation that PanK activity increased in *Ehdpck1* and *Ehdpck2* gs strains. Although none of the intermediates, 4′-phosphopantothenate, 4′-phosphopantothenoyl-L-cysteine, pantetheine 4-phosphate, and dephospho-CoA was measured in our metabolomics analysis, it is conceivable that these metabolites accumulated intracellularly as a consequence of lack of *Eh*DPCK and upregulation of *Eh*PanK. These data also reinforce allosteric feedback of *Eh*PanK by the CoA regulatory network of CoA biosynthesis, which was demonstrated in our previous study (Nurkanto et al., 2018).

Finally, together with our previous report on *Eh*PanK, which catalyzes the first step of CoA biosynthesis (Nurkanto et al., 2018), the two key enzymes of CoA biosynthesis, *Eh*PanK and *Eh*DPCKs have been proven to be attractive and rational targets for the development of anti-amebic agents. However, *Eh*PanK and *Eh*DPCKs need to be further validated for suitability as drug target in the future by screening chemical and microbial libraries to discover specific inhibitors.

## Conclusion

We have demonstrated that *Eh*DPCK is the important enzyme involved in CoA biosynthesis and its repression causes significant disturbance in the level of some intermediates in polyamine, hexose, and nucleic acid metabolisms in the proliferative trophozoite stage of *E. histolytica*. *Eh*DPCK2 plays a predominant role compared to *Eh*DPCK1 in trophozoites stage. Notable differences in kinetic parameters of DPCK between *Entamoeba* and its host suggest that amebic DPCK is the rational target for the development of anti-amebic agents.

## Author Contributions

AN, GJ, YN, MS, TH, and TN designed the experiments and analyzed the data. AN, GJ, TY, YN, and THI performed the experiments. AN, TH, and TN wrote the manuscript. TN conceived the project and supervised the study. All authors reviewed the results and approved the final version of the manuscript.

## Conflict of Interest Statement

The authors declare that the research was conducted in the absence of any commercial or financial relationships that could be construed as a potential conflict of interest.

## Footnotes

1 http://www.brenda-enzymes.info/

2 http://www.ncbi.nlm.nih.gov/

3 http://tree.bio.ed.ac.uk/software/figtree/

4 http://www.sbg.bio.ic.ac.uk/phyre2

5 https://www.genome.jp/kegg

6 http://amoebadb.org/amoeba/

7 http://tritrypdb.org/tritrypdb/

## Supplementary Material

FIGURE S1 Alignment of DPCK protein sequences from *E. histolytica* and other organisms. The sequences were aligned using Muscle program (Edgar, 2004) in SeaView package version 4.6.1 (Gouy et al., 2010). Identical residues in these conserved domains are denoted by asterisks (^∗^). The GXXXXGKS/T p-loop consensus sequence, which is an ATP/GTP-binding motif, is shown in gray background. The lysine residues are highlighted with white letters on black background. Human DPCK is located at the carboxyl terminus of a fused bifunctional enzyme of phosphopantetheine adenylyltransferase and dephospho-CoA kinase (COASY), which catalyzes the last two steps of CoA biosynthesis.

FIGURE S2 Phylogenetic tree of dephospho-CoA Kinase (DPCK) proteins from *E. histolytica* and other organisms. Optimal ML tree inferred by RAxML program with LG + Ã4 model was shown from 112 DPCK positions were used in this analysis. The numbers at the nodes represent the bootstrap values as a percentage of 100 replicates. The scale bar indicates 0.3 substitutions at each amino acid position.

FIGURE S3 Expression and purification of recombinant *Eh*DPCK1 **(A,B)** and *Eh*DPCK2 **(C,D)**. **(A,C)** Protein samples of recombinant *Eh*DPCK1 **(A)** and *Eh*DPCK2 **(C)** at each step of purification were subjected to 15% SDS-PAGE under reducing conditions and then stained with Coomassie Brilliant Blue R250. **(B,D)** Immunoblot analysis of purified recombinant *Eh*DPCK1 **(B)** and *Eh*DPCK2 **(D)** using anti-His antibody.

FIGURE S4 Optimum pH of the recombinant *Eh*DPCK1 **(A)** and *Eh*DPCK2 **(B)**. Enzymes activities were measured at 37°C at the various pHs indicated in the figure. Assays were performed as described in Section “Experimental procedures,” with 50 ng of *Eh*DPCK1 or *Eh*DPCK2 recombinant enzyme, 15 mM HEPES, 20 mM NaCl, 10 mM MgCl_2_, 1 mM EGTA, 0.02% Tween-20, 0.1 mg/mL β-globulins, 100 μM ATP, and 200 μM dephospho-CoA at 37°C. Data are shown in mean ± SE of three replicates.

FIGURE S5 Inhibition activities of the *Eh*DPCK1 **(A)** and *Eh*DPCK2 **(B)** by CoA, acetyl-CoA, and malonyl-CoA. The assays were carried out three times independently. Data are shown in mean ± SE.

FIGURE S6 Growth kinetic of *E. histolytica* transformants during 96 h incubation in BI-S-33 medium observed using WST1 **(A,B)** and the cellular fractionation and immunoblot analysis of *Eh*DPCK2 **(C)**. Data are shown in mean of absorbance 450 nm ± SE of three replicates **(A,B)** as described in Section “Materials and Methods.” Figure **(C)** is immunoblot analysis using anti-HA monoclonal antibody, anti-CPBF1, and anti-CS1 polyclonal antisera, where CPBF1 and CS1 serve as a control for organelle and cytosolic proteins, respectively. Whole lysate of control strain was also subjected to analysis.

FIGURE S7 Relative levels of gene transcripts encoding *Ehdpck1* **(A)** and *Ehdpck2***(B)** and its related and unrelated genes in CoA biosynthesis pathway. Data were normalized against *RNA polymerase II*, and are shown in percentage relative to the transcript level of each gene in psAP2 control. NADHK, Tom-40, and CPBF2 are corresponding to NAD(H) kinase (EHI_151920), mitosomal membrane protein-Tom40 (EHI_104420), and cysteine protease binding protein 2 (EHI_087660), respectively. Data are shown in mean ± SE. Statistical comparison is made by Student’s *t*-test (^∗^*P* < 0.05, ^∗∗^*P* < 0.01).

FIGURE S8 Metabolic profiles of amino acids (ornithine, glutamate, and methionine), polyamines, and associated metabolites affected by *Ehdpck1* gene silencing. Broken lines indicate genes and their encoded enzymes are likely absent in the genome. Labels and abbreviations are as in Figure 4. Data are shown in mean ± SE. Statistical comparison is made by Student’s *t*-test (^∗^*P* < 0.05).

FIGURE S9 Metabolic profiles of hexose phosphates, glycogen metabolism, chitin biosynthesis, and associated metabolism affected by *Ehdpck1* gene silencing. Labels and abbreviations are as in Figure 5. Data are shown in mean ± SE.

FIGURE S10 Metabolic profiles of purine metabolism affected by *Ehdpck2* gene silencing. Broken lines indicate genes whose encoding enzymes known to catalyze the steps are likely absent in the genome. Labels and abbreviations are as in Figure 6. Data are shown in mean ± SE. Statistical comparison is made by Student’s *t*-test (^∗^*P* < 0.05).

FIGURE S11 Relative levels of *dpck1* and *2* gene transcripts in *E. invadens* encystation stage. Data was generated from our previous publication (De Cádiz et al., 2013).

TABLE S1 Sequence data of dephospho-CoA kinase (DPCK) used in phylogenetic analysis.

TABLE S2 Protein concentrations, activities, specific activities, percentage yield, and fold purification of recombinant EhDPCK.

TABLE S3 Metabolite profiles of *Ehdpck1* and *Ehdpc2* gene silencing strain obtained from CE-MS analysis. All data are shown as means ± SE from three independent biological replicates, and for each biological replicate, two technical replicates were made.
