# Supplementary material for: Biochemical, Metabolomic, and Genetic Analyses of Dephospho Coenzyme A Kinase Involved in Coenzyme A Biosynthesis in the Human Enteric Parasite Entamoeba histolytica
Source: Front Microbiol. 2018 Nov 30;9:2902. doi: 10.3389/fmicb.2018.02902 (PMC6284149; doi:10.3389/fmicb.2018.02902)
Supplement: FIGURE S1 — Alignment of DPCK protein sequences from E. histolytica and other organisms. The sequences were aligned using Muscle program (Edgar, 2004) in SeaView package version 4.6.1 (Gouy et al., 2010). Identical residues in these conserved domains are denoted by asterisks (∗). The GXXXXGKS/T p-loop consensus sequence, which is an ATP/GTP-binding motif, is shown in gray background. The lysine residues are highlighted with white letters on black background. Human DPCK is located at the carboxyl terminus of a fused bifunctional enzyme of phosphopantetheine adenylyltransferase and dephospho-CoA kinase (COASY), which catalyzes the last two steps of CoA biosynthesis. [file Data_Sheet_1.PDF]

Fig. S1

```
                                                            50                                                     100
E._histolytica_1    --MKKIFVIG ITGSICSGKS RLCKALQS-K GIKTIDSDTI GHDVILQED- VKKEIKESFG ---EEVFNE- EGQVDRKRLS DVVFTNKKQL KKLNEIMWNS
E._histolytica_2    ----MVFVIG ITGRICSGKS TLSKTLIK-R KIPVIDCDKL GHELYQKGSI VYNDFIKLFG ---KEVVGV- DGSIDRKKLS ELVFSNPQNV KIISDLTWPA
Dictyostelium       --MSKLIKIG LTGGIASGKS TILGYLKE-M NIKCIDADKF GHMVYQKGRP SYNKIIQEFG ---QDIINQN DQSIDRSKLG PIVFSDPLKM KQLTNIVWPE
Saccharomyces       -----MLVVG LTGGIACGKS TVSRRLRDKY KLPIVDADKI ARQVVEPGQN AYDQIVLYFK DKIPNLLLE- DGHLNREALG KWVFSHKEDL QALNGITHPA
Homo*               -----LYVIG LTGISGSGKS SIAQRLKG-L GAFVIDSDHL GHRAYAPGGP AYQPVVEAFG ---TDILHK- DGIINRKVLG SRVFGNKKQL KILTDIMWPI
Drosophila_2        -----MFIVA VTGGIATGKS TISKVFER-Q GIPVIDADKI AREIVEPGQP CWRQIREVFG ---DEVLLP- SKEINRAVLG KMIFEDKELR GKLNKITHPT
Arabidopsis         -----MRIVG LTGGIASGKS TVSNLFKA-S GIPVVDADVV ARDVLKKGSG GWKRVVAAFG ---EEILLP- SGEVDRPKLG QIVFSSDSKR QLLNKLMAPY
Trypanosoma         -----MLLVG LTGGIACGKS TVSLLLKESH HIVVVDSDLV VRELQRPFMP CTRKIARRW- ---PNCVDPQ SGEVNRAALG SIIFSDPSAR RALARIMNFP
Emiliania           -----MRIVG LTGGIACGKS AVSTAAAS-W GLRVIDMDEI ARRVVCPGLP AYTDIVAAFG ---DGALLP- SGELNRTEVG RIAFSDREKR RALNAATHRP
Hydrogenobacter     -----MLKVG LTGNIGCGKS TVARMFME-L GAYTFDADAI IRTFYQEKGE VYRKVVEAFG ---EGILDK- EGNIDRKKLA DLVFLDINKL RLLESITHKA
Hirschia            -----MIILG LTGSIGMGKS TTANIFLD-A GIPVYDSDAA VHALYQSGGK AVDKLEDAF- ---PGVKNQ- EGSIDRQKLS AYVLKDSAAM KKLEAIVHPL
Geobacter           -----MRIIG LTGGIASGKS TVTRILER-L GTAVIDADQL AREAVVPGTA GHRAIVETFG ---PAVLLP- DGAIDRPALG RIIFADPVAR GKLEAITHPA
Escherichia         ----MRYIVA LTGGIGSGKS TVANAFAN-L GINVIDADII ARQVVEPGAP ALHAIADHFG ---ANMIAA- DGTLQRRALR ERIFANPEEK NWLNDLLHPL
Chlamydia           MSEKKVLMIG ITGGIASGKS TVVAMIKE-A GYQVIDADQV VHQLQEKGGR LYEALKQAFG ---NEILKE- DGDLNRKKLS ELVFSNPSHM ATSSAIQNQI
Eggerthella         ----MAKRIF ITGGIGSGKS TLLDFLRS-Q GVAAVEADKV GHEVLTFADT -KAELAEKFG ---NDIFDD- EGNVVRSALA AKAFVSPEMT ALLDSVTLKR
Dehalococcoides     -----MKIVG ITGGIGSGKT TVCRYLKE-L GVNIIDADEI GHRVLQNKGI -RTKITDVFG ---NEVMNP- DGNINRKILG ELVFGYPERL ERLNKITHPL
Anabaena            ---MKKHLIG LTGGIATGKS TVANYLVTAY NLPMLDADVY ARDAVSVGSP IITQISQRYG ---KQILLP- DGNLNRSQLG EIIFNQPEER HWVENLIHPY
Clostridium         -----MLRIG LTGSIGCGKS SLSNILKK-Y NIPIIDADIK GREIYEDKEL -LKAIEDAFG ---NSVINK- DGTLNRKNLG KIVFSDDDKL EKLNSLTHPV
                                 * **  **                    *
```

```
                                                                       150                                                    200
E._histolytica_1    IENKIKEQI- ----QQLEI- -QGE--KIVA VEAALLIR-T NWMSW-MDMI WVTTVSPEIA IARMTAGR-G MTREECINRM KNQPSSREYA RYADILFDTR
E._histolytica_2    IYTLLQERI- ----KVLEK- -EGH--PIVG VEAALLIR-A NW-SI-VNVI WETRIDEAET IKRLKTRN-G LSEEQAKKRI QSQPTQEEYD NKSQVQINTM
Dictyostelium       MKELILNEF- ----KEIETT CPND--KIVV LEAAVLIE-A GFNEM-VDLI WVTQVPREVA IERLKTRN-N LSEVDACKRI DSQLTNEERE KYANLVFKTN
Saccharomyces       IRYAMFKEI- ----GYYYL- -KGY--RMCV LDVPLLFE-G NLDSI-CGVT VSVICTQELQ LERLMTRNPE LSEEDAKNRL NSQMSTEERM ARSDYILQNN
Homo                IAKLAREEM- ----DRAVA- -EGK--RVCV IDAAVLLE-A GWQNL-VHEV WTAVIPETEA VRRIVERD-G LSEAAAQSRL QSQMSGQQLV EQSHVVLSTL
Drosophila_2        IHRKIFWQV- ----CKLLV- -TGH--AWIV LDLPLLFE-T GVLMDFIHKI VCVTCDSDKQ LERLIARN-E LSESEARHRV DSQMPLDKKC EKSHFVIDNN
Arabidopsis         ISSGIFWEI- ----LKQWA- -SGA--KVIV VDIPLLFE-V KMDKW-TKPI VVVWVSQETQ LKRLMERD-G LSEEDARNRV MAQMPLDSKR SKADVVIDNN
Trypanosoma         IFRATMKMVI GLWWQSLRQQ LRGQGPLLVV LDVPLLYE-S NIYTWLVDRV VVVSCSEEQQ VERMAKRN-G LTREQALQRI NAQMPISEKC KRADRVIHNE
Emiliania           MLMQLLAEL- ----AAALF- -GGE--AAVL IDAPLLFE-T SLHWL-CCTT LVVAA---TQ LERLRARD-G FSAEEAEKRI AAQMPLAEKC ARADAVIDNE
Hydrogenobacter     LYERLEEEF- ----RRLP-- -QD---AIAI VEASLLVE-K GTYKN-YDRL IVVYAPYEVC KERAIKS--G FSSEDFERRW KNQMPPEEKL KYADFVIDNS
Hirschia            VFETRLKFL- ----DEHKA- -KGT--DIVV FDIPLLFE-T GSDAF-VDKI LVVTAPAEIQ KQRVLDRE-G MTEEKFSAIL AKQMPDKQKR ERADFIIDTS
Geobacter           IAALAEQKL- ----AELRR- -RGE--RVAF YVAPLLIE-A GATGR-VDEI WVVYVDRETQ LARLTARD-G ITRHEAEQRL AAQMPMEEKA ARGAVVIDNR
Escherichia         IQQETQHQI- ----QQATS- ------PYVL WVVPLLVE-N SLYKK-ANRV LVVDVSPETQ LKRTMQRD-D VTREHVEQIL AAQATREARL AVADDVIDNN
Chlamydia           IKEELAAER- ----DQLA-- -QSQ--TVIF MDIPLLIE-L GYQDW-FDAI WLVYVDAQTQ LRRLMARN-H LREVDAKKRL SSQLSIEEKR PYASLVIDNS
Eggerthella         VYAESLRQL- ----DNLA-- -QTH--DFVA LEMAILDGRD DFYKN-ADVV LAVVTTPEVR IARLVGR--G LTEEDARNRL KNQVPDSKRE AIADVVFTNN
Dehalococcoides     IEQAISSLL- ----EEYRQ- -KGI--KSVA IEAPLLVE-A GWLKL-VNEV WLITAPKESI FKRLHSRM-G LSREQAIARI QSQATDNERL KYASVVVNNN
Anabaena            VRNCFTQAI- ----NELSA- ------NTLI LVIPLLFE-A GLENL-VGEI WVVSCSPQQQ QQRLIQRN-N LTNEQAVARI SSQLSIAEKV ARADVVLDNS
Clostridium         IRRMINEDL- ----DEYEK- -RGE--KMAV IDAALLLE-A GFMSM-LDTI IVVTCSEPVQ LQRVILRD-N CSEEDAMGRI KSQMPQEEKV KYAEFVVDNS
                                                          *                             *                     *
```

```
                                                                   250
E._histolytica_1   FNRGTLERIF NREINAFLID FNENKF---- ---------- ---------- --------
E._histolytica_2   GTIEETATLI NKEVDKLLEQ LKITLN---- ---------- ---------- --------
Dictyostelium      DDYEITKNKV QNEINNLLNN YNK------- ---------- ---------- --------
Saccharomyces      STLVDLYEQI ESVVKKIQPS KLRTVLEYFP PFGAVSASSI VMSRLLMKKL QNKKSSAV
Homo               WEPHITQRQV EKAWALLQKR IPKTHQALD- ---------- ---------- --------
Drosophila_2       GSVEEAESSA MSIYNLMRDS KQHWLNRISF LGLFLIVGFT IYMLLKVFNR LPESWQM-
Arabidopsis        GSLDDLHQQF EKVLIEIRRP LTWIEFWRSR QGAFSVLGSV ILGLSVCKQL KIGS----
Trypanosoma        ESLSELEHSV ADTVAWMQQQ SGGRVAFALS GALAVGVSLG AVVVYCCLRI VF------
Emiliania          GTRDELAARA ATILDAAGAI AGQTP----- ---------- ---------- --------
Hydrogenobacter    GSLESTQKQV KKVYRELLNL VH-------- ---------- ---------- --------
Hirschia           LGLDSAEKNV YQIIQSLIQL NESED----- ---------- ---------- --------
Geobacter          GEPSELERQV LMLWDRHCRA GD-------- ---------- ---------- --------
Escherichia        GAPDAIASDV ARLHAHYLQL ASQFVSQEKP ---------- ---------- --------
Chlamydia          GDMETLRKQV HKALEQLSKP Y--------- ---------- ---------- --------
Eggerthella        GTIEDFENEI ALWWESFKNE AAK------- ---------- ---------- --------
Dehalococcoides    CRFEDLKACV QLLAKERLEL A--------- ---------- ---------- --------
Anabaena           STLESLLQQI DLVMKRVDTN LI-------- ---------- ---------- --------
Clostridium        GTLDQLAEEA DELIRKLEEK IEK------- ---------- ---------- --------
```

Fig. S2

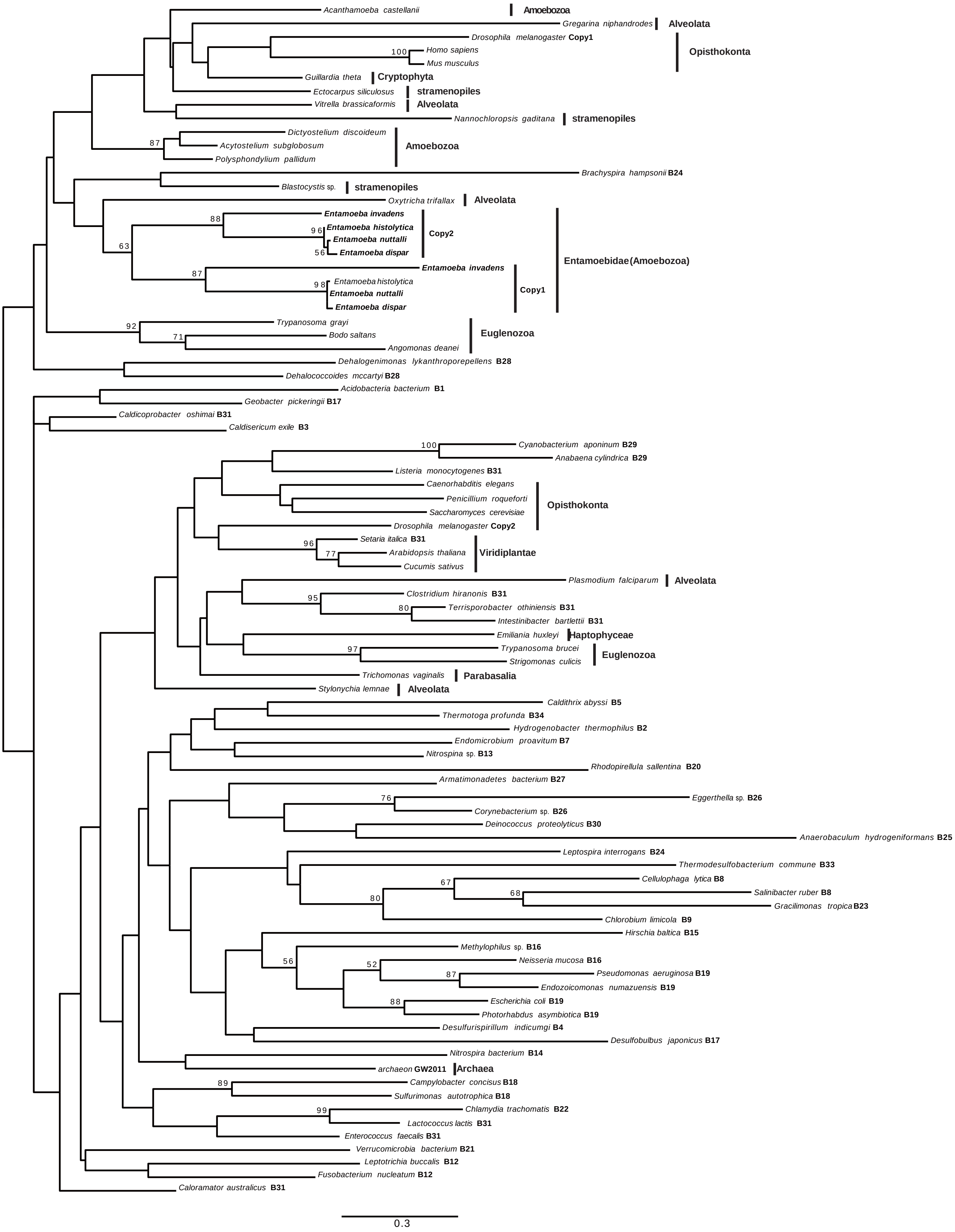
Acanthamoeba castellanii
Amoebozoa
Gregarina niphandrodes
Alveolata
Drosophila melanogaster Copy1
100
Homo sapiens
Mus musculus
Opisthokonta
Guillardia theta
Cryptophyta
Ectocarpus siliculosus
stramenopiles
Vitrella brassicaformis
Alveolata
Nannochloropsis gaditana
stramenopiles
Dictyostelium discoideum
87
Acytostelium subglobosum
Polysphondylium pallidum
Amoebozoa
Brachyspira hampsonii B24
Blastocystis sp.
stramenopiles
Oxytricha trifallax
Alveolata
88
Entamoeba invadens
96
Entamoeba histolytica
Entamoeba nuttalli
56
Entamoeba dispar
Copy2
63
87
Entamoeba invadens
98
Entamoeba histolytica
Entamoeba nuttalli
Entamoeba dispar
Copy1
Entamoebidae (Amoebozoa)
92
Trypanosoma grayi
71
Bodo saltans
Angomonas deanei
Euglenozoa
Dehalogenimonas lykanthroporepellens B28
Dehalococcoides mccartyi B28
Acidobacteria bacterium B1
Geobacter pickeringii B17
Caldicoprobacter oshimai B31
Caldisericum exile B3
100
Cyanobacterium aponinum B29
Anabaena cylindrica B29
Listeria monocytogenes B31
Caenorhabditis elegans
Penicillium roqueforti
Saccharomyces cerevisiae
Opisthokonta
Drosophila melanogaster Copy2
96
Setaria italica B31
77
Arabidopsis thaliana
Cucumis sativus
Viridiplantae
Plasmodium falciparum
Alveolata
95
Clostridium hiranonis B31
80
Terrisporobacter othiniensis B31
Intestinibacter bartlettii B31
Emiliania huxleyi
Haptophyceae
97
Trypanosoma brucei
Strigomonas culicis
Euglenozoa
Trichomonas vaginalis
Parabasalia
Stylonychia lemnae
Alveolata
Caldithrix abyssi B5
Thermotoga profunda B34
Hydrogenobacter thermophilus B2
Endomicrobium proavitum B7
Nitrospina sp. B13
Rhodopirellula sallentina B20
Armatimonadetes bacterium B27
76
Eggerthella sp. B26
Corynebacterium sp. B26
Deinococcus proteolyticus B30
Anaerobaculum hydrogeniformans B25
Leptospira interrogans B24
Thermodesulfobacterium commune B33
67
Cellulophaga lytica B8
68
Salinibacter ruber B8
80
Gracilimonas tropica B23
Chlorobium limicola B9
Hirschia baltica B15
Methylophilus sp. B16
56
52
Neisseria mucosa B16
87
Pseudomonas aeruginosa B19
Endozoicomonas numazuensis B19
88
Escherichia coli B19
Photorhabdus asymbiotica B19
Desulfurispirillum indicumgi B4
Desulfobulbus japonicus B17
Nitrospira bacterium B14
archaeon GW2011
Archaea
89
Campylobacter concisus B18
Sulfurimonas autotrophica B18
99
Chlamydia trachomatis B22
Lactococcus lactis B31
Enterococcus faecalis B31
Verrucomicrobia bacterium B21
Leptotrichia buccalis B12
Fusobacterium nucleatum B12
Caloramator australicus B31
0.3

Fig. S3

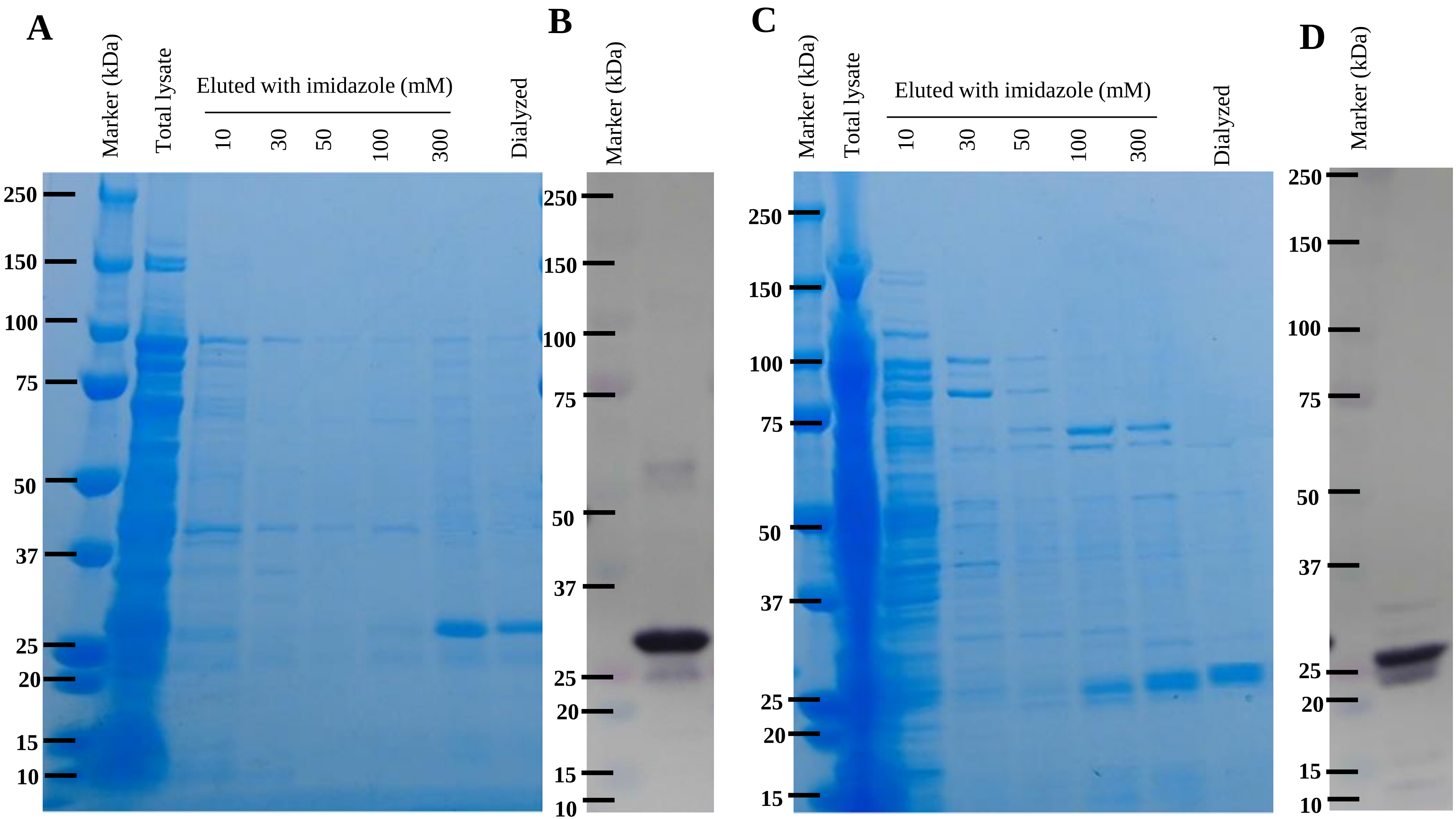
A
Marker (kDa)
Total lysate
Eluted with imidazole (mM)
10
30
50
100
300
Dialyzed
250
150
100
75
50
37
25
20
15
10
B
Marker (kDa)
250
150
100
75
50
37
25
20
15
10
C
Marker (kDa)
Total lysate
Eluted with imidazole (mM)
10
30
50
100
300
Dialyzed
250
150
100
75
50
37
25
20
15
D
Marker (kDa)
250
150
100
75
50
37
25
20
15
10

Fig. S4

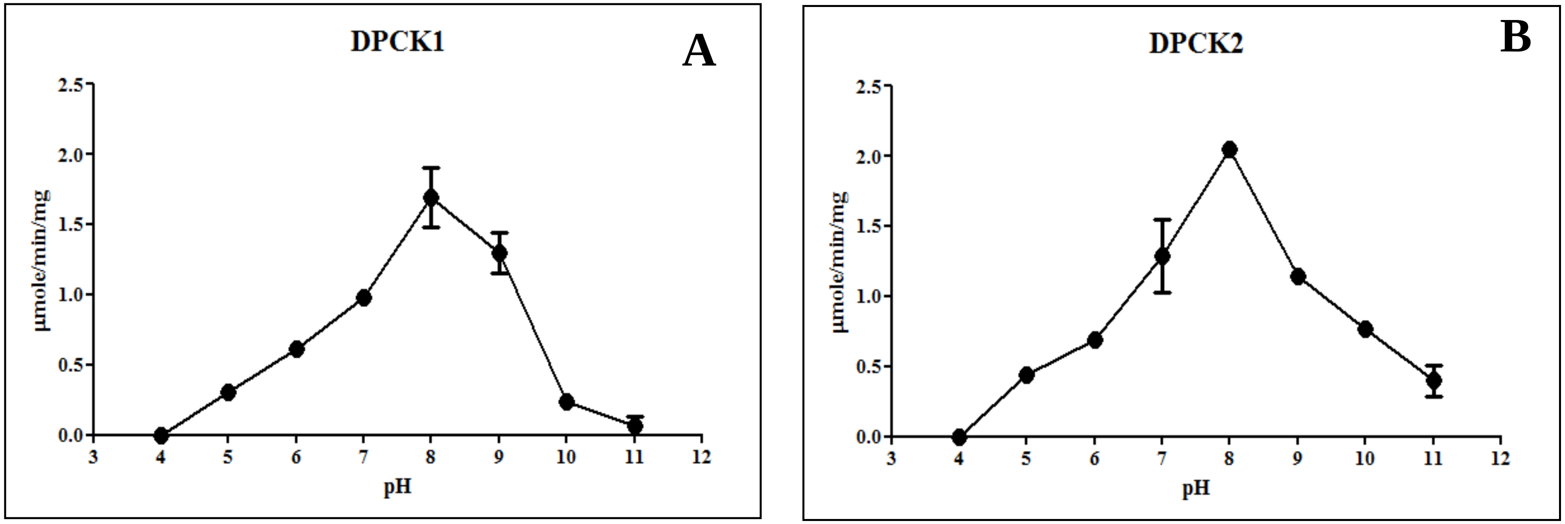
DPCK1
A
2.5
2.0
1.5
1.0
0.5
0.0
μmole/min/mg
3
4
5
6
7
8
9
10
11
12
pH
DPCK2
B
2.5
2.0
1.5
1.0
0.5
0.0
μmole/min/mg
3
4
5
6
7
8
9
10
11
12
pH

Fig. S5

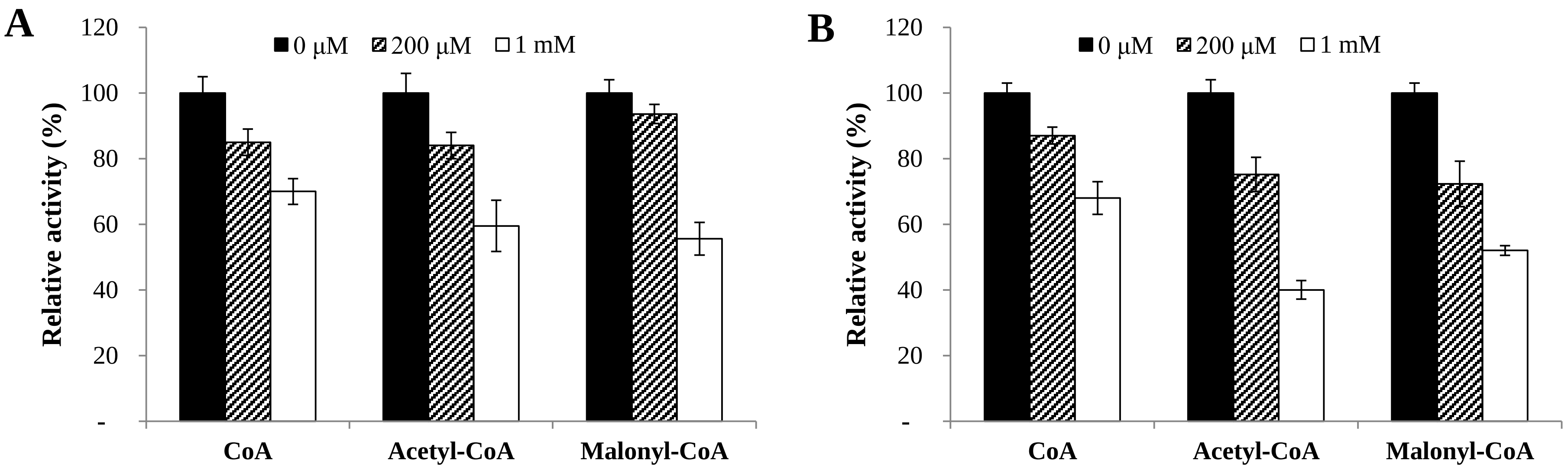
A
120
100
80
60
40
20
-
Relative activity (%)
0 µM
200 µM
1 mM
CoA
Acetyl-CoA
Malonyl-CoA
B
120
100
80
60
40
20
-
Relative activity (%)
0 µM
200 µM
1 mM
CoA
Acetyl-CoA
Malonyl-CoA

Fig. S6

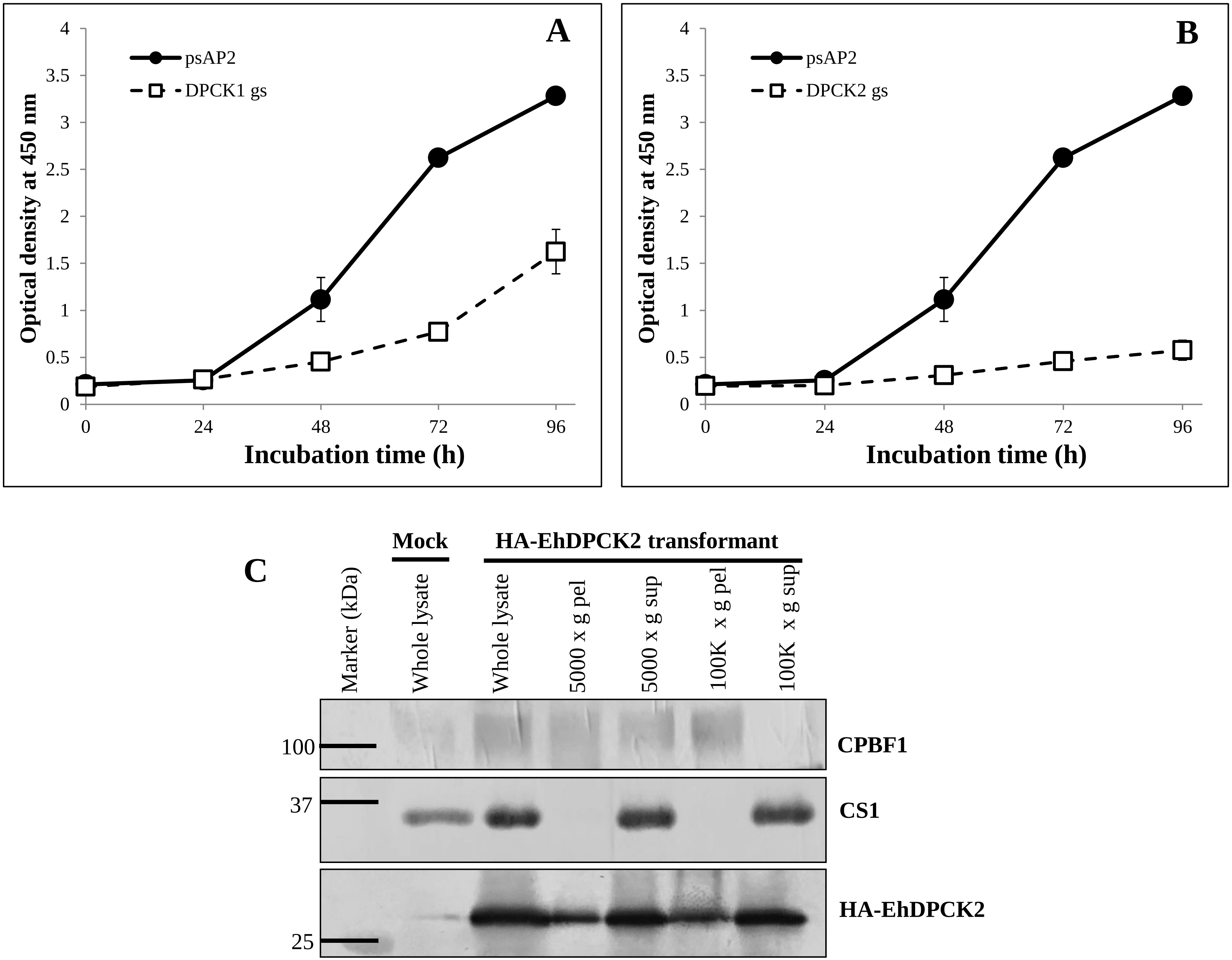
A
psAP2
DPCK1 gs
Optical density at 450 nm
Incubation time (h)
0
24
48
72
96
0
0.5
1
1.5
2
2.5
3
3.5
4
B
psAP2
DPCK2 gs
Optical density at 450 nm
Incubation time (h)
C
Mock
HA-EhDPCK2 transformant
Marker (kDa)
Whole lysate
Whole lysate
5000 x g pel
5000 x g sup
100K x g pel
100K x g sup
100
37
25
CPBF1
CS1
HA-EhDPCK2

Fig. S7

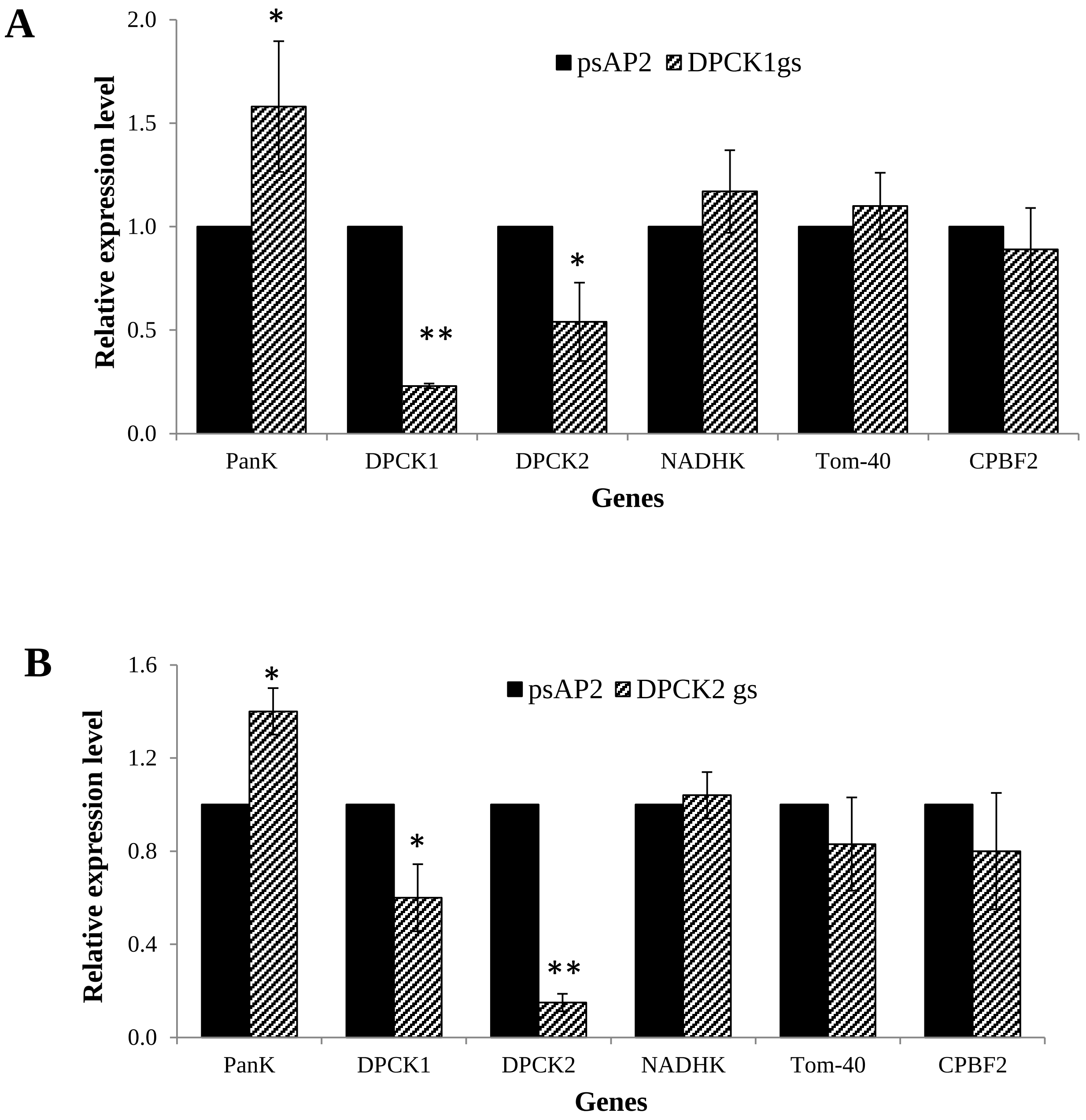
A
B
psAP2
DPCK1gs
DPCK2 gs
Relative expression level
2.0
1.5
1.0
0.5
0.0
1.6
1.2
0.8
0.4
*
**
PanK
DPCK1
DPCK2
NADHK
Tom-40
CPBF2
Genes

Fig. S8

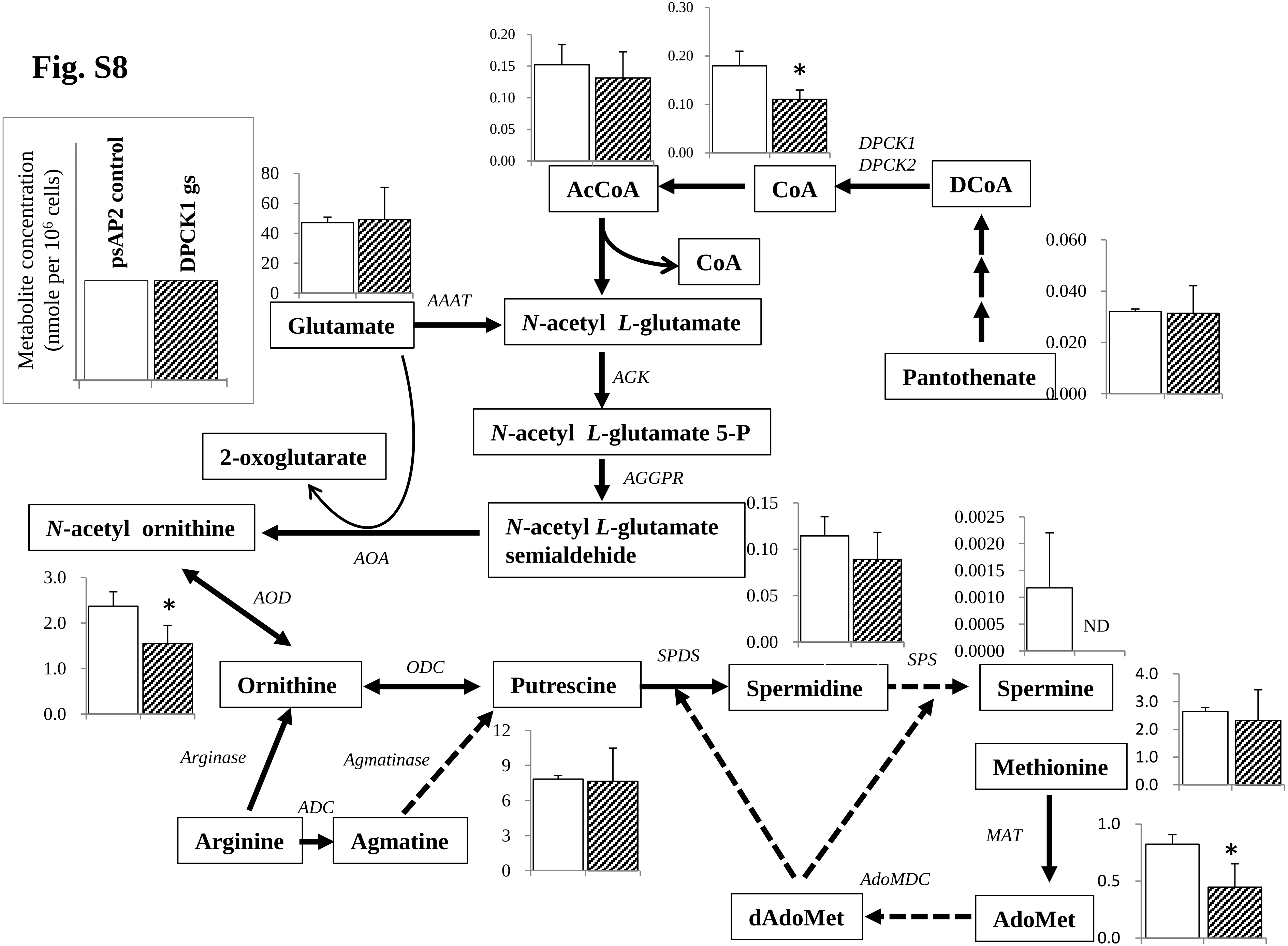

Metabolite concentration (nmole per 10^6 cells)
psAP2 control
DPCK1 gs
AcCoA
CoA
DCoA
DPCK1
DPCK2
CoA
Glutamate
AAAT
N-acetyl L-glutamate
AGK
N-acetyl L-glutamate 5-P
AGGPR
N-acetyl L-glutamate semialdehide
Pantothenate
2-oxoglutarate
N-acetyl ornithine
AOA
AOD
Ornithine
ODC
Putrescine
SPDS
Spermidine
SPS
Spermine
ND
Methionine
MAT
AdoMet
AdoMDC
dAdoMet
Arginase
Agmatinase
ADC
Arginine
Agmatine
*

Fig. S9

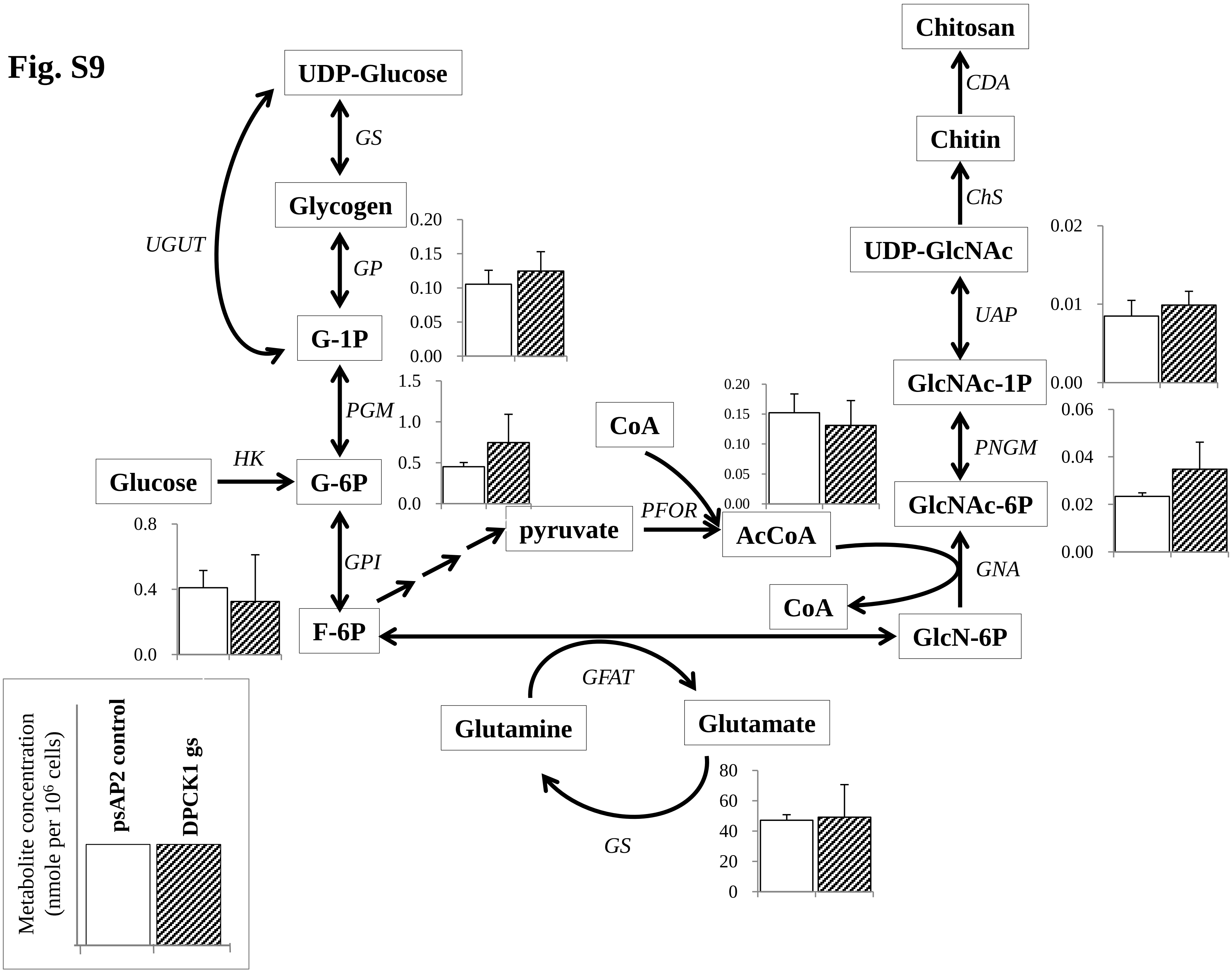

Chitosan
CDA
Chitin
ChS
UDP-GlcNAc
UAP
GlcNAc-1P
PNGM
GlcNAc-6P
GNA
GlcN-6P
CoA
AcCoA
PFOR
pyruvate
CoA
UDP-Glucose
GS
Glycogen
GP
G-1P
UGUT
PGM
G-6P
HK
Glucose
GPI
F-6P
GFAT
Glutamine
Glutamate
GS
Metabolite concentration
(nmole per 10^6 cells)
psAP2 control
DPCK1 gs

Fig. S10

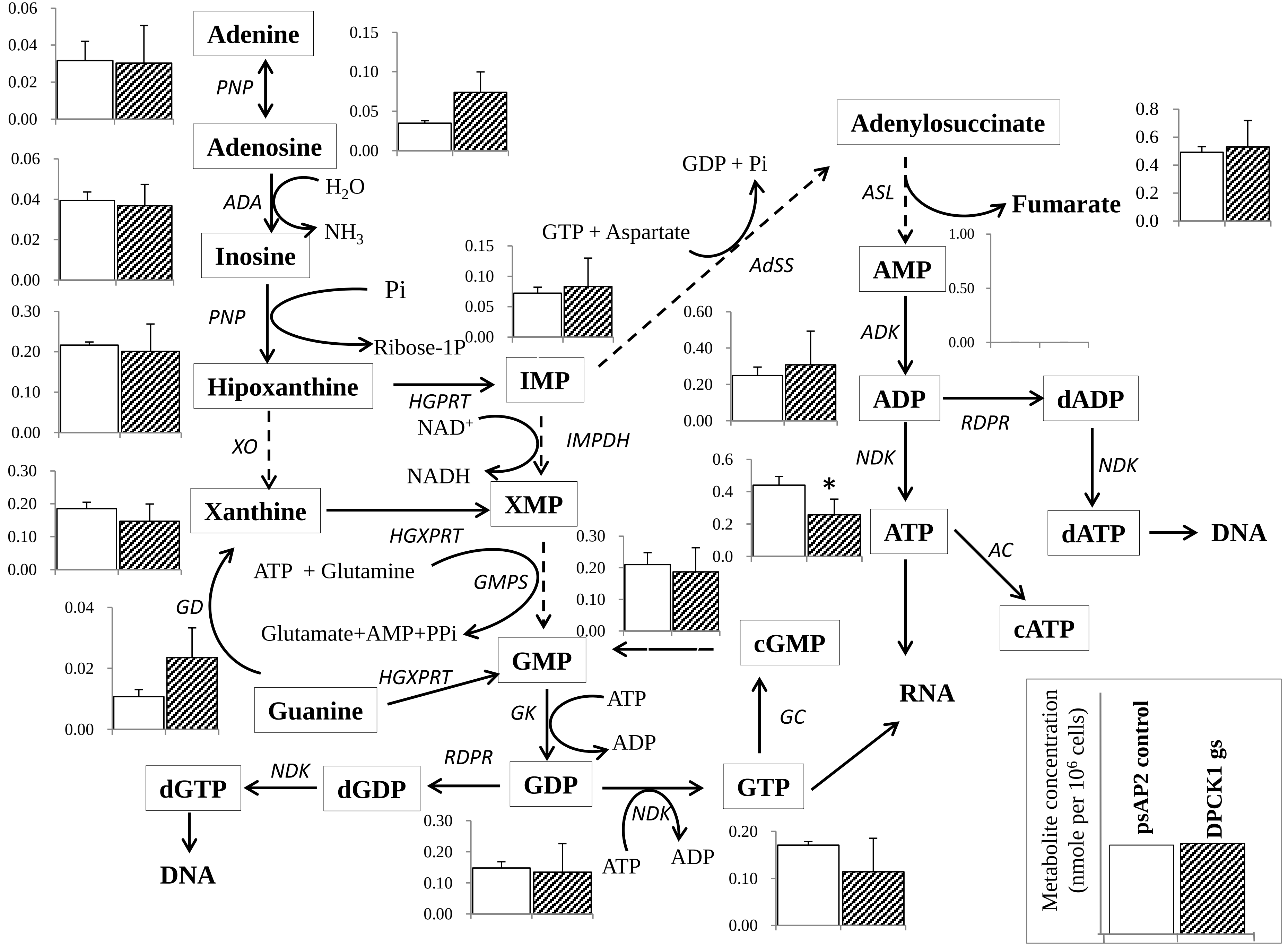

Adenine
PNP
Adenosine
ADA
H_2O
NH_3
Inosine
PNP
Pi
Ribose-1P
Hipoxanthine
HGPRT
IMP
XO
NAD+
NADH
IMPDH
Xanthine
HGXPRT
XMP
ATP + Glutamine
GMPS
Glutamate+AMP+PPi
GD
Guanine
HGXPRT
GMP
GK
ATP
ADP
GDP
RDPR
dGDP
NDK
dGTP
DNA
NDK
ATP
ADP
GTP
GC
cGMP
GTP + Aspartate
GDP + Pi
AdSS
Adenylosuccinate
ASL
Fumarate
AMP
ADK
ADP
RDPR
dADP
NDK
NDK
ATP
AC
cATP
dATP
DNA
RNA
*
Metabolite concentration (nmole per 10^6 cells)
psAP2 control
DPCK1 gs

Fig. S11

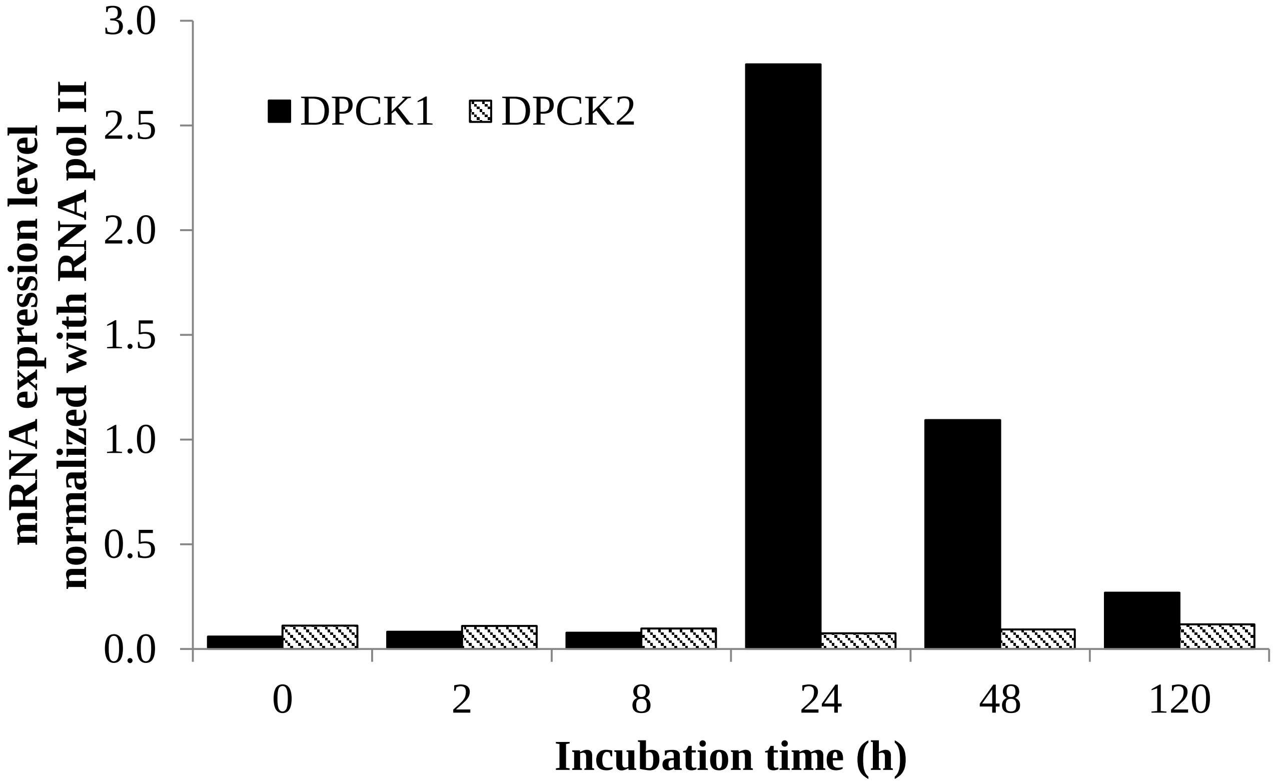

3.0
2.5
2.0
1.5
1.0
0.5
0.0
DPCK1
DPCK2
mRNA expression level
normalized with RNA pol II
0
2
8
24
48
120
Incubation time (h)
