# Supplementary material for: Biochemical, Metabolomic, and Genetic Analyses of Dephospho Coenzyme A Kinase Involved in Coenzyme A Biosynthesis in the Human Enteric Parasite Entamoeba histolytica
Source: Front Microbiol. 2018 Nov 30;9:2902. doi: 10.3389/fmicb.2018.02902 (PMC6284149; doi:10.3389/fmicb.2018.02902)
Supplement: TABLE S1 — Sequence data of dephospho-CoA kinase (DPCK) used in phylogenetic analysis. [file Data_Sheet_2.PDF]

Table S1. Sequence data of dephospho-CoA kinase (DPCK) used in phylogenetic analysis.

**Taxonomic group**	**Group ID**			
**EUKARYOTA**				
Alveolata	E1	*Plasmodium falciparum*<br>XP_001348589	*Gregarina niphandrodes*<br>XP_011129458	*Vitrella brassicaformis*<br>CCMP3155<br>CEL98141
		Oxytricha trifallax<br>EJY78014	*Stylonychia lemnae*<br>CDW86686	
Amoebozoa	E2	*Entamoeba histolytica* _1<br>XP_648971	*Entamoeba histolytica* _2<br>XP_655761	*Entamoeba nuttalli* _1<br>XP_008856749
		Entamoeba nuttalli- 2<br>XP_008856164	*Entamoeba dispar*- 1<br>XP_001737160	*Entamoeba dispar*- 2<br>XP_001741223
		Entamoeba invadens- 1<br>XP_004261640	*Entamoeba invadens*- 2<br>XP_004262031	*Polysphondylium pallidum*<br>EFA79885
		Acytostelium subglobosum- LB1<br>XP_012759261	*Dictyostelium discoideum*-<br>AX4	*Acanthamoeba castellanii*<br>str.-Neff
Apusozoa	E3			
Breviatea	E4			
Centroheliozoa	E5			
Cryptophyta	E6	*Guillardia theta* CCMP2712<br>XP_005819820		
Euglenozoa	E7	*Strigomonas culicis*<br>EPY31869	*Trypanosoma grayi*<br>XP_009306665	*Trypanosoma brucei*<br>XP_845194
		Angomonas deanei<br>EPY26666	*Bodo saltans*<br>CUG88465	

Fornicata	E8			
Glaucocystophyceae	E9			
Haptophyceae	E10	*Emiliania huxleyi* -CCMP1516<br>XP_005782461		
Heterolobosea	E11			
Jakobida	E12			
Katablepharidophyta	E13			
Malawimonadidae	E14			
Opisthokonta	E15	*Saccharomyces cerevisiae*<br>NP_010482	*Penicillium roqueforti*<br>CDM29792	*Drosophila melanogaster*<br>NP_649692
		Drosophila melanogaster<br>NP_647985	*Caenorhabditis elegans*<br>CBY25196	*Mus musculus*<br>EDL03879
		Homo_sapiens<br>AAL50813		
Oxymonadida	E16			
Parabasalia	E17	*Trichomonas vaginalis* -G3<br>XP_001579493		
Rhizaria	E18			
Rhodophyta (red algae)	E19			
stramenopiles (heterokonts)	E20	*Nannochloropsis gaditana*<br>EWM28160	*Blastocystis* sp._NandII<br>OAO12384	*Ectocarpus siliculosus*<br>CBN76945

Viridiplantae (green plants)	E21	*Arabidopsis thaliana* NP_180318	*Cucumis sativus* XP_011652920

ARCHAEA

Euryarchaeota		A1	
Nanoarchaeota		A2	
TACK group	Thaumarchaeota	A3	
	Aigarchaeota	A4	
	Crenarchaeota	A5	
	Korarchaeota	A6	
Unclassified Archaea		A7	*Archaeon* GW2011-AR10 AJF60481

BACTERIA

Acidobacteria	B1	*Acidobacteria bacterium* -Mor1 ANM29893
Aquificae	B2	*Hydrogenobacter thermophilus* WP_012963603
Caldiserica	B3	Caldisericum_exile WP_014453443

Chrysiogenetes		B4	*Desulfurispirillum indicumgi* WP_013505067	
Deferribacteres		B5	*Caldithrix abyssi* WP_006930425	
Dictyoglomi		B6		
Elusimicrobia	Endomicrobia	B7	*Endomicrobium proavitum* WP_052570597	
FCB group	Bacteroidetes	B8	*Salinibacter ruber* WP_011405495	*Cellulophaga lytica* WP_013622844
	Chlorobi	B9	*Chlorobium limicola* WP_059138936	
	Fibrobacteres	B10		
	Gemmatimonadetes	B11		
Fusobacteria		B12	*Fusobacterium nucleatum* WP_020789018	*Leptotrichia buccalis* WP_015770131
Nitrospinae/Tectomicrobia group	Nitrospinae	B13	*Nitrospina* sp.-SCGC_AAA799_C22	
Nitrospirae		B14	*Nitrospira bacterium*- SG8-3 KPK22519	
Proteobacteria	Alphaproteobacteria	B15	*Hirschia baltica* WP_015828962	
	Betaproteobacteria	B16	*Methylophilus* sp. Leaf414 WP_055867614	*Neisseria mucosa* WP_003748310
	Deltaproteobacteria	B17	*Geobacter pickeringii* WP_039744153	*Desulfobulbus japonicus* WP_051309098
	Epsilonproteobacteria	B18	*Campylobacter concisus* WP_021083770	*Sulfurimonas autotrophica* WP_013327920

	Gammaproteobacteria	B19	*Photorhabdus asymbiotica* WP_012777299	*Endozoicomonas numazuensis*	*Escherichia coli* WP_001584165
			Pseudomonas aeruginosa WP_014603303		
PVC group	Planctomycetes	B20	*Rhodopirellula sallentina* WP_008684538		
	Verrucomicrobia	B21	*Verrucomicrobia bacterium*- LP2 WP_024808731		
	Chlamydiae	B22	*Chlamydia trachomatis* CRH93337		
Rhodothermaeota		B23	*Gracilimonas tropica* WP_020403094		
Spirochaetes		B24	*Brachyspira hampsonii*- 30599 ELV05054	*Leptospira interrogans* WP_001181517	
Synergistetes		B25	*Anaerobaculum hydrogeniformans*		
Terrabacteria group	Actinobacteria	B26	*Eggerthella* sp._CAG:298 CDD60299	*Corynebacterium* sp.-JZ16 ANE03904	
	Armatimonadetes	B27	*Armatimonadetes bacterium*- CSP1-3		
	Chloroflexi	B28	*Dehalococcoides mccartyi* WP_011309681	*Dehalogenimonas lykanthroporepellens*	
	Cyanobacteria	B29	*Anabaena cylindrica* WP_015217232	*Cyanobacterium aponinum* WP_015218179	
	Deinococcus-Thermus	B30	*Deinococcus proteolyticus* WP_013614615		
	Firmicutes	B31	*Lactococcus lactis* WP_010905495	*Enterococcus faecalis* WP_010713834	*Setaria italica* XP_004968727
			Intestinibacter bartlettii WP_039905943	*Clostridium hiranonis* WP_040410665	*Terrisporobacter othiniensis*

			Caldicoprobacter oshimai WP_025747566	*Caloramator australicus* WP_008907570	*Listeria monocytogenes* WP_009926374
	Tenericutes	B32			
Thermodesulfobacteria		B33	*Thermodesulfobacterium commune*		
Thermotogae		B34	*Thermotoga profunda* WP_041083684		

Table S2. Protein concentrations, activities, specific activities, percentage yield, and fold purification of recombinant EhDPCK.

**Enzymes**	**Purification step**	**Protein concentration (mg)**	**Activity (µmole/min)**	**Specific activity (µmole/min/mg)**	**Yield (%)**	**Purification (fold)**
EhDPCK1	Lysate	2.32	2.09	0.90	100	-
	Eluate	0.36	0.77	2.13	36.7	2.4
EhDPCK2	Lysate	4.48	3.09	0.69	100	-
	Eluate	0.35	0.89	2.54	28.8	3.7

Table S3. Metabolite profiles of *Ehdpck1* and *Ehdpck2* gene silencing obtained from CE-MS analysis.

Ehdpck1 gs

concentration in nmole/10^6 cell

No.	Compound	Control strain		EhDPCK1 gs		t-test (control vs EhDPCK1 gs)		Fold changes
		Mean	SD	Mean	SD	*p* -value	-	
1	Glucose 1-phosphate	0.105	0.020	0.125	0.028	0.339	▵	1.19
2	Glucose 6-phosphate	0.451	0.053	0.746	0.344	0.183	▵	1.66
3	Fructose 6-phosphate	0.410	0.105	0.326	0.285	0.615	▼	0.79
4	Fructose 1,6-diphosphate	0.047	0.005	0.016	0.020	0.054	▼	0.35
5	Dihydroxyacetonephosphate	ND	-	0.022	0.043	-	▵	
6	DL-Glyceraldehyde 3-phosphate	0.122	0.011	0.099	0.114	0.705	▼	0.80
7	3-Phosphoglycerate	0.031	0.003	0.025	0.005	0.128	▼	0.80
8	Phosphoenolpyruvate	0.040	0.008	0.035	0.014	0.530	▼	0.86
9	Pyruvate	0.142	0.028	0.103	0.050	0.249	▼	0.72
10	Lactate	0.251	0.039	0.281	0.023	0.321	▵	1.12
11	Σ glycolysis (G1P～PEP)	1.206	0.162	1.393	0.379	0.423	▵	1.16
12	Glycerol 3-phosphate	0.137	0.019	0.297	0.183	0.178	▵	2.17
13	Acetyl CoA	0.152	0.032	0.131	0.041	0.479	▼	0.86
14	Citrate	0.153	0.011	0.091	0.012	0.001	▼	0.59
15	α-Ketoglutaric acid	0.606	0.155	0.364	0.198	0.131	▼	0.60
16	Succinate	0.323	0.041	0.339	0.151	0.853	▵	1.05
17	Fumarate	0.492	0.039	0.529	0.189	0.724	▵	1.08
18	Malate	0.288	0.025	0.304	0.217	0.897	▵	1.05
19	Ribulose 5-phosphate	0.060	0.007	0.071	0.035	0.562	▵	1.20
20	Ribose 5-phosphate	0.020	0.004	0.018	0.008	0.685	▼	0.90
21	D-Sedoheptulose 7-phosphate	0.076	0.014	0.163	0.076	0.104	▵	2.15
22	Erythrose 4-phosphate	ND	-	0.072	0.145	-	▵	
23	ΣPPP	0.155	0.026	0.325	0.228	0.235	▵	2.09
24	Methionine Sulfoxide	0.535	0.019	0.537	0.279	0.991	▵	1.00
25	S-Adenosyl-L-methionine	0.823	0.084	0.446	0.226	0.037	▼	0.54
26	Putrescine	7.835	0.319	7.639	2.845	0.900	▼	0.97
27	Spermidine	0.114	0.021	0.089	0.029	0.233	▼	0.78
28	Spermine	0.001	0.001	ND	-	-	▼	
29	S-Adenosyl-L-homocysteine	0.011	0.000	0.012	0.004	0.591	▵	1.10
30	Homocysteine	0.011	0.009	0.023	0.004	0.146	▵	2.11
31	Homocystine	0.049	0.002	0.033	0.011	0.065	▼	0.68
32	Cystathionine	0.078	0.004	0.074	0.030	0.782	▼	0.94
33	O-Succinyl-L-homoserine	0.137	0.006	0.137	0.004	0.971	▼	1.00
34	Homoserine	0.232	0.045	0.204	0.067	0.538	▼	0.88
35	Taurine	0.055	0.007	0.055	0.023	0.978	▵	1.01
36	O-Phospho-L-serine	0.012	0.003	0.012	0.002	0.842	▼	0.97
37	Cystine	0.009	0.002	0.008	0.014	0.909	▼	0.91

38	Glutathione, reduced form	0.007	0.012	0.004	0.005	0.707	▼	0.55
39	Glutathione, oxidized form	0.007	0.012	0.006	0.011	0.915	▼	0.85
40	N-Methyl-Arginine	0.003	0.001	0.001	0.001	0.138	▼	0.42
41	ADMA	0.039	0.004	0.040	0.014	0.963	△	1.01
42	SDMA	0.003	0.002	0.002	0.001	0.373	▼	0.65
43	GSH/GSSG	1.025	-	0.231	-	-	▼	0.23
44	GSH/(GSH+2xGSSG)[%]	33.894	-	55.172	63.397	-	△	1.63
45	GSH+2xGSSG	0.020	0.035	0.015	0.024	0.843	▼	0.75
46	SAM/SAH	78.386	8.975	43.200	33.608	0.125	▼	0.55
47	Ophthalmic acid	0.020	0.001	0.017	0.005	0.380	▼	0.86
48	Ornithine	2.371	0.317	1.553	0.394	0.029	▼	0.66
49	Citrulline	1.514	0.020	1.534	0.337	0.914	△	1.01
50	Creatine	0.043	0.003	0.045	0.021	0.820	△	1.06
51	Creatinine	0.016	0.001	0.015	0.005	0.723	▼	0.93
52	Hydroxyproline	0.009	0.016	ND	-	-	▼	
53	Glycine	4.898	0.587	6.161	1.856	0.274	△	1.26
54	Alanine	3.413	0.051	5.591	3.230	0.270	△	1.64
55	Serine	0.807	0.040	0.724	0.153	0.364	▼	0.90
56	Threonine	0.339	0.022	0.296	0.028	0.074	▼	0.87
57	Valine	16.202	0.647	17.731	5.509	0.619	△	1.09
58	Isoleucine	8.558	1.616	8.804	3.069	0.897	△	1.03
59	Leucine	29.182	2.340	31.262	11.431	0.745	△	1.07
60	Lysine	10.880	0.340	10.637	2.511	0.860	▼	0.98
61	Arginine	0.542	0.022	0.519	0.215	0.842	▼	0.96
62	Histidine	3.002	0.191	2.627	0.718	0.382	▼	0.88
63	Tyrosine	1.703	0.104	1.845	0.749	0.732	△	1.08
64	Phenylalanine	1.750	0.133	2.274	1.733	0.589	△	1.30
65	Tryptophan	1.035	0.044	1.070	0.451	0.887	△	1.03
66	Methionine	2.633	0.152	2.320	1.099	0.611	▼	0.88
67	Cysteine	0.086	0.107	0.078	0.092	0.919	▼	0.90
68	Proline	16.548	0.999	17.005	4.420	0.853	△	1.03
69	Glutamine	3.478	0.126	4.579	2.000	0.352	△	1.32
70	Glutamate	47.194	3.584	49.184	21.465	0.866	△	1.04
71	Asparagine	1.557	0.106	2.180	1.279	0.402	△	1.40
72	Aspartic acid	0.340	0.025	0.405	0.127	0.385	△	1.19
73	Adenine	0.032	0.010	0.030	0.020	0.917	▼	0.96
74	Guanine	0.011	0.002	0.024	0.030	0.450	△	2.21
75	Cytosine	0.007	0.000	0.005	0.002	0.164	▼	0.79
76	Uracil	0.096	0.006	0.107	0.033	0.553	△	1.11
77	Adenosine	0.035	0.003	0.074	0.086	0.432	△	2.11
78	Guanosine	0.044	0.006	0.069	0.073	0.547	△	1.56
79	Cytidine	0.063	0.008	0.054	0.021	0.443	▼	0.85
80	Uridine	0.886	0.085	0.687	0.378	0.374	▼	0.77
81	Inosine	0.039	0.004	0.037	0.011	0.670	▼	0.93

82	AMP	0.295	0.074	0.300	0.147	0.953	△	1.02
83	GMP	0.210	0.038	0.187	0.076	0.628	▼	0.89
84	CMP	0.017	0.005	0.016	0.004	0.734	▼	0.93
85	UMP	0.098	0.016	0.110	0.034	0.583	△	1.12
86	IMP	0.072	0.010	0.083	0.047	0.676	△	1.15
87	cAMP	0.927	0.134	0.530	0.198	0.025	▼	0.57
88	cGMP	0.530	0.092	0.251	0.083	0.013	▼	0.47
89	cCMP	0.196	0.030	0.117	0.038	0.028	▼	0.60
90	ADP	0.249	0.046	0.308	0.286	0.712	△	1.23
91	GDP	0.148	0.020	0.135	0.092	0.795	▼	0.91
92	CDP	0.018	0.003	0.019	0.009	0.939	△	1.02
93	UDP	0.041	0.005	0.032	0.007	0.088	▼	0.77
94	ATP	0.441	0.053	0.258	0.096	0.025	▼	0.59
95	GTP	0.171	0.008	0.114	0.071	0.211	▼	0.67
96	CTP	0.029	0.004	0.023	0.006	0.124	▼	0.77
97	UTP	0.093	0.005	0.058	0.010	0.003	▼	0.63
98	dATP	0.007	0.002	0.001	0.002	0.021	▼	0.17
99	Hypoxanthine	0.216	0.008	0.201	0.068	0.678	▼	0.93
100	Xanthine	0.185	0.020	0.147	0.052	0.254	▼	0.79
101	NAD	0.134	0.052	0.103	0.084	0.573	▼	0.77
102	NADP	0.094	0.028	0.083	0.069	0.797	▼	0.89
103	ATP+ADP+AMP+Adenosine	1.019	0.174	0.940	0.579	0.809	▼	0.92
104	(ATP+1/2ADP)/(ATP+ADP+AMP)	0.577	0.025	0.483	0.042	0.015	▼	0.84
105	Uric acid	ND	-	0.011	0.022	-	△	
106	Pantothenate	0.032	0.001	0.031	0.011	0.895	▼	0.98
107	L-Carnitine	0.009	0.001	0.007	0.002	0.263	▼	0.83
108	N-Propionyl-CoA	0.003	0.001	0.003	0.002	0.444	△	1.30
109	Glucosamine 6-phosphate	0.012	0.002	0.011	0.008	0.865	▼	0.94
110	N-Acetyl-D-glucosamine	0.162	0.009	0.170	0.035	0.687	△	1.05
111	N-Acetyl-D-glucosamine 6-phosphate	0.023	0.001	0.035	0.011	0.137	△	1.49
112	N-Acetyl-α-D-glucosamine 1-phosphate	0.008	0.002	0.010	0.002	0.389	△	1.16
113	Carnosine	0.033	0.025	0.049	0.038	0.538	△	1.49
114	Glycolate	0.120	0.025	0.180	0.038	0.051	△	1.51
115	S-Methyl-L-cysteine	0.013	0.003	0.017	0.007	0.296	△	1.36

* ▼ = decreased compared to control psAP2
△ = increased compared to control psAP2

Ehdpck2 gs

concentration in nmole/10^6 cell

No.	Compound	Control strain		EhDPCK2 gs		t-test (control vs EhDPCK2 gs)		Fold changes
		Mean	SD	Mean	SD	*p* -value	-	
1	Glucose 1-phosphate	0.064	0.027	0.047	0.020	0.535	▼	0.73
2	Glucose 6-phosphate	0.734	0.120	0.465	0.113	0.119	▼	0.63
3	Fructose 6-phosphate	0.258	0.014	0.177	0.066	0.161	▼	0.68
4	Fructose 1,6-diphosphate	0.062	0.006	0.046	0.016	0.214	▼	0.73
5	Dihydroxyacetonephosphate	0.110	0.011	0.111	0.026	0.935	▵	1.01
6	3-Phosphoglycerate	0.040	0.008	0.045	0.016	0.681	▵	1.12
7	Phosphoenolpyruvate	0.054	0.009	0.063	0.025	0.608	▵	1.17
8	Pyruvate	ND	-	0.150	0.099	-	▵	
9	Lactate	0.547	0.005	1.442	1.391	0.381	▵	2.64
10	Σglycolysis (G1P～PEP)	1.322	0.144	0.954	0.256	0.132	▼	0.72
11	Glycerol 3-phosphate	0.120	0.009	0.118	0.101	0.971	▼	0.98
12	Acetyl-CoA	0.103	0.014	0.046	0.007	0.071	▼	0.45
13	Citrate	0.093	0.007	0.165	0.067	0.203	▵	1.77
14	α-Ketoglutaric acid	0.381	0.058	0.419	0.097	0.625	▵	1.10
15	Succinate	0.412	0.056	0.382	0.168	0.791	▼	0.93
16	Fumarate	0.243	0.027	0.173	0.058	0.168	▼	0.71
17	Malate	0.159	0.031	0.170	0.053	0.794	▵	1.07
20	Ribulose 5-phosphate	0.054	0.008	0.038	0.018	0.280	▼	0.70
21	Ribose 5-phosphate	0.023	0.003	0.023	0.004	0.866	▵	1.03
22	D-Sedoheptulose 7-phosphate	0.090	0.004	0.084	0.023	0.671	▼	0.93
23	ΣPPP	0.167	0.016	0.145	0.046	0.504	▼	0.87
24	Methionine Sulfoxide	0.282	0.042	0.154	0.075	0.093	▼	0.55
25	S-Adenosyl-L-methionine	0.458	0.034	0.200	0.022	0.023	▼	0.44
26	Putrescine	4.396	0.165	2.956	0.129	0.012	▼	0.67
27	Spermidine	0.039	0.003	0.017	0.007	0.021	▼	0.44
28	Spermine	0.001	0.002	0.001	0.002	0.871	▼	0.77
29	S-Adenosyl-L-homocysteine	0.009	0.001	0.008	0.001	0.724	▼	0.96
30	Homocysteine	0.016	0.022	0.016	0.014	0.989	▵	1.02
31	Homocystine	0.012	0.017	0.050	0.020	0.125	▵	4.09
32	Cystathionine	0.053	0.031	0.056	0.017	0.901	▵	1.07
33	O-Succinyl-L-homoserine	0.331	0.060	0.336	0.010	0.937	▵	1.01
34	Homoserine	0.141	0.025	0.157	0.023	0.536	▵	1.11
35	Taurine	0.073	0.007	0.062	0.023	0.511	▼	0.85
36	O-Phospho-L-serine	0.014	0.001	0.017	0.001	0.150	▵	1.20
37	Cystine	0.004	0.006	0.004	0.007	0.992	▵	1.02
38	N-Methyl-Arg	0.001	0.001	0.002	0.002	0.739	▵	1.51
39	ADMA	0.021	0.001	0.015	0.005	0.165	▼	0.71
40	SDMA	0.002	0.001	0.002	0.001	0.768	▼	0.87
41	SAM/SAH	53.775	2.370	24.623	3.927	0.002	▼	0.46
42	Ophthalmic acid	0.016	0.001	0.020	0.020	0.789	▵	1.23

43	Glu	33.185	1.931	18.818	4.052	0.014	▼	0.57
44	Ornithine	1.318	0.014	0.774	0.203	0.043	▼	0.59
45	Citrulline	1.314	0.201	1.078	0.409	0.455	▼	0.82
46	Creatine	0.024	0.004	0.019	0.006	0.352	▼	0.80
47	Creatinine	0.008	0.002	0.006	0.001	0.465	▼	0.84
48	Glycine	2.885	0.257	2.022	0.648	0.138	▼	0.70
49	Alanine	2.444	0.520	2.881	1.015	0.573	△	1.18
50	Serine	0.508	0.014	0.409	0.245	0.554	▼	0.80
51	Threonine	0.213	0.005	0.214	0.114	0.993	△	1.00
52	Valine	8.110	0.028	4.922	1.316	0.052	▼	0.61
53	Isoleucine	4.239	0.057	2.420	0.517	0.024	▼	0.57
54	Leucine	14.040	2.019	8.415	3.242	0.097	▼	0.60
55	Lysine	6.052	1.264	4.426	1.486	0.293	▼	0.73
56	Arginine	0.337	0.021	0.357	0.155	0.843	△	1.06
57	Histidine	1.698	0.224	1.393	0.510	0.432	▼	0.82
58	Tyrosine	0.895	0.051	0.560	0.159	0.054	▼	0.63
59	Phenylalanine	0.854	0.021	0.668	0.282	0.371	▼	0.78
60	Tryptophan	0.594	0.051	0.253	0.139	0.037	▼	0.43
61	Methionine	1.272	0.036	0.717	0.218	0.043	▼	0.56
62	Cysteine	0.067	0.020	0.073	0.084	0.907	△	1.10
63	Proline	11.291	0.683	6.438	2.211	0.050	▼	0.57
64	Glutamine	2.009	0.122	1.360	0.485	0.137	▼	0.68
65	Glutamate	33.185	1.931	18.818	4.052	0.014	▼	0.57
66	Asparagine	0.837	0.058	0.652	0.098	0.078	▼	0.78
67	Aspartic acid	0.187	0.000	0.192	0.133	0.952	△	1.03
68	Adenine	0.051	0.006	0.023	0.001	0.098	▼	0.46
69	Guanine	0.011	0.001	0.006	0.011	0.545	▼	0.58
70	Cytosine	0.003	0.005	0.004	0.003	0.986	△	1.02
71	Uracil	0.106	0.022	0.101	0.021	0.844	▼	0.96
72	Adenosine	0.011	0.001	0.008	0.006	0.473	▼	0.71
73	Guanosine	0.020	0.012	0.013	0.014	0.566	▼	0.62
74	Cytidine	0.038	0.004	0.043	0.017	0.718	△	1.11
75	Uridine	0.465	0.067	0.275	0.052	0.089	▼	0.59
76	Inosine	0.041	0.014	0.024	0.005	0.308	▼	0.58
77	AMP	0.057	0.012	0.044	0.049	0.695	▼	0.77
78	GMP	0.078	0.033	0.034	0.033	0.271	▼	0.44
79	CMP	ND	-	0.008	0.015	-	△	
80	UMP	0.017	0.023	0.017	0.030	0.982	△	1.03
81	IMP	0.034	0.009	0.011	0.020	0.187	▼	0.33
82	cAMP	0.163	0.050	0.026	0.004	0.159	▼	0.16
83	cGMP	0.076	0.024	ND	-	-	▼	
84	cCMP	0.050	0.017	0.042	0.008	0.645	▼	0.84
85	ADP	0.107	0.009	0.063	0.034	0.146	▼	0.59
86	GDP	0.095	0.002	0.048	0.011	0.014	▼	0.50

87	CDP	0.008	0.004	0.008	0.007	0.954	▼	0.96
88	TDP	ND	-	0.002	0.003	-	▵	
89	UDP	0.012	0.004	0.021	0.020	0.534	▵	1.71
90	ATP	0.348	0.000	0.148	0.037	0.011	▼	0.43
91	GTP	0.162	0.014	0.089	0.027	0.030	▼	0.55
92	CTP	0.029	0.002	0.027	0.012	0.780	▼	0.92
93	UTP	0.084	0.001	0.058	0.023	0.179	▼	0.68
94	dGTP	ND	-	0.002	0.004	-	▵	
95	Hypoxanthine	0.188	0.022	0.046	0.018	0.021	▼	0.25
96	Xanthine	0.120	0.006	0.055	0.028	0.050	▼	0.46
97	NADP	0.051	0.007	0.030	0.013	0.107	▼	0.60
98	ATP+ADP+AMP+Adenosine	0.523	0.022	0.263	0.126	0.064	▼	0.50
99	(ATP+1/2ADP)/(ATP+ADP+AMP)	0.784	0.024	0.737	0.111	0.549	▼	0.94
100	Pantothenate	0.030	0.001	0.022	0.004	0.049	▼	0.71
101	L-Carnitine	0.005	0.001	0.006	0.002	0.403	▵	1.31
102	Malonyl CoA	0.007	0.010	ND	-	-	▼	
103	N-Propionyl CoA	0.001	0.002	ND	-	-	▼	
104	NAD	0.080	0.020	0.049	0.024	0.217	▼	0.61
105	UDP-glucose	0.064	0.003	ND	-	0.023	▼	
106	Glucosamine 6-phosphate	0.012	0.001	ND	-	-	▼	
107	N-Acetyl-D-glucosamine	0.216	0.043	0.209	0.069	0.901	▼	0.97
108	N-Acetyl-D-glucosamine 6-phosphate	0.030	0.001	0.021	0.000	0.009	▼	0.71
109	N-Acetyl-α-D-glucosamine 1-phosphate	0.012	0.002	0.011	0.003	0.658	▼	0.90
110	Carnosine	0.023	0.021	0.031	0.019	0.707	▵	1.35
111	Glycolate	0.246	0.063	0.338	0.040	0.243	▵	1.37
112	S-Methyl-L-cysteine	0.012	0.017	0.006	0.011	0.715	▼	0.51

* ▼ = decreased compared to control psAP2
▵ = increased compared to control psAP2
